# Single‐Entity Electrochemistry of N‐Doped Graphene Oxide Nanostructures for Improved Kinetics of Vanadyl Oxidation

**DOI:** 10.1002/smll.202405220

**Published:** 2024-11-16

**Authors:** Maida Aysla Costa de Oliveira, Marc Brunet Cabré, Christian Schröder, Hugo Nolan, Filippo Pota, James A. Behan, Frédéric Barrière, Kim McKelvey, Paula E. Colavita

**Affiliations:** ^1^ School of Chemistry Trinity College Dublin Dublin 2 Ireland; ^2^ Univ Rennes CNRS Institut des Sciences Chimiques de Rennes – UMR 6226 Rennes F‐35000 France; ^3^ MacDiarmid Institute for Advanced Materials and Nanotechnology School of Chemical and Physical Sciences Victoria University of Wellington Wellington 6012 New Zealand

**Keywords:** graphene oxide, nanoelectrochemistry, n‐doping, SECCM, vanadyl oxidation

## Abstract

N‐doped graphene oxides (GO) are nanomaterials of interest as building blocks for 3D electrode architectures for vanadium redox flow battery applications. N‐ and O‐functionalities have been reported to increase charge transfer rates for vanadium redox couples. However, GO synthesis typically yields heterogeneous nanomaterials, making it challenging to understand whether the electrochemical activity of conventional GO electrodes results from a sub‐population of GO entities or sub‐domains. Herein, single‐entity voltammetry studies of vanadyl oxidation at N‐doped GO using scanning electrochemical cell microscopy (SECCM) are reported. The electrochemical response is mapped at sub‐domains within isolated flakes and found to display significant heterogeneity: small active sites are interspersed between relatively large inert sub‐domains. Correlative Raman‐SECCM analysis suggests that defect densities are not useful predictors of activity, while the specific chemical nature of defects might be a more important factor for understanding oxidation rates. Finite element simulations of the electrochemical response suggest that active sub‐domains/sites are smaller than the mean inter‐defect distance estimated from Raman spectra but can display very fast heterogeneous rate constants >1 cm s^−1^. These results indicate that N‐doped GO electrodes can deliver on intrinsic activity requirements set out for the viable performance of vanadium redox flow battery devices.

## Graphical abstract

Isolated N‐doped graphene oxide flakes are characterized using scanning electrochemical cell microscopy (SECCM) to investigate their performance in V(V/IV) reactions. The response of sub‐domains is heterogeneous even within a single flake. However, very fast charge transfer is observed at defect sites, indicating that these nanomaterials hold great potential as building blocks for high‐performing electrodes in redox flow battery applications.

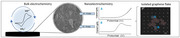

## 1 Introduction

Vanadium redox flow batteries (VRFB) hold promise in energy storage due to their high capacity, long service life, and deep discharge capabilities.^[^ ^1^ ^]^ VRFB consist of two tanks separated by an ion‐exchange membrane with porous carbon electrodes at which charge/discharge reactions occur.^[^ ^1^, ^2^ ^]^ Conventional graphitic carbons are often employed because of low cost, good conductivity, and physical properties.^[^ ^3^ ^]^ However, the kinetics of the relevant VO_2_^+^/VO^2+^ and V^3+^/V^2+^ redox couples are generally sluggish at these surfaces, limiting overall performance;^[^ ^3^, ^4^ ^]^ therefore, there is great interest in improving charge transfer kinetics at carbons for VRFB.

**Figure 1:**
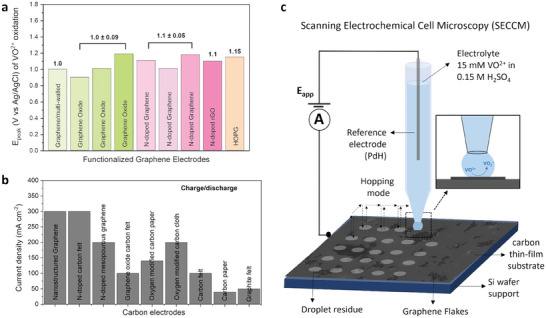
a) Peak potential values for VO^2+^/VO_2_^+^ oxidation from literature for a range of carbon materials. b) Current densities obtained with VRFB devices using graphene‐based electrodes; references relevant to panels (a,b) are listed in Table S1 (Supporting Information). c) Schematic of the SECCM experimental configuration used to characterize drop‐cast GO flakes.

Several groups have reported improved performances upon functionalization of carbons with oxygen‐ and nitrogen‐containing moieties.^[^ ^4^, ^5^ ^]^ This has been attributed to a range of effects including increased electrochemically active surface area (ECSA), improved wettability, and enhanced charge transfer kinetics.^[^ ^3^, ^6^ ^]^ **Figure** **1**a (also see Table S1, Supporting Information) shows a comparison of reported peak potential values for the VO^2+^ oxidation at various carbons: N‐/O‐modified carbons generally perform better than purely graphitic carbons. This is also observed with VRFB devices for instance, in Figure 1b, by comparing current densities obtained with conventional carbons versus heteroatom‐modified carbons. N‐functionalities have been proposed to improve conductivity and modulate electron transfer kinetics.^[^ ^6^, ^7^ ^]^ However, improved performance, especially in the case of oxidized moieties, can be the result of complex and contrasting effects, as functionalization can increase roughness, wettability, and ultimately ECSA while, conversely, also resulting in degradation of the intrinsic activity and conductivity of carbons.^[^ ^5^, ^6^, ^8^ ^]^

Work from our group using model carbon thin films showed that pyridinic/pyrrolic functionalities significantly improve the intrinsic heterogeneous rate constant (*k* ^0^) and electrochemical reversibility of the VO_2_^+^/VO^2+^ faradaic response.^[^ ^6f^ ^]^ A 100‐fold increase in *k* ^0^ to ca. 10^−4^ cm s^−1^ relative to a N‐free graphitic surface was indeed observed. However, a recent analysis^[^ ^4d^ ^]^ suggests that, for operational VRFB devices, an effective charge transfer constant $k_{eff}^{0}$ of 10^−2^ cm s^−1^ is desirable to minimize kinetic contributions to overpotential. This is a difficult target to meet through improvements of intrinsic activity alone, that will most likely require optimization of high ECSA electrodes based on carbon nanomaterials with intrinsic heterogeneous rate constants better than 10^−4 ^cm s^−1^.^[^ ^4d^ ^]^ Therefore, an in‐depth understanding of intrinsic activity and achievable performances of nanomaterials remains crucial to meet VRFB requirements through design and optimization of carbon electrode architectures.

Graphene oxide (GO) and N‐doped GO are promising nanomaterials for VRFB applications, as illustrated in Figure 1a,b.^[^ ^8^, ^9^ ^]^ GO and N‐doped GO can be obtained via low‐cost methods such as electrochemical/chemical exfoliation that are suited to applications at scale^[^ ^9^, ^10^ ^]^ but typically yield heterogeneous nanomaterials.^[^ ^11^ ^]^ Therefore, it is unclear whether the ensemble responses observed with conventional GO electrodes are the result of a sub‐population of GO entities or of specific sites/domains within individual GO entities.^[^ ^12^ ^]^ Interestingly, sub‐domains significantly smaller than the lateral size of GO flakes are well known to often determine photoemission, conductance, and friction properties;^[^ ^13^ ^]^ however, the potential impact of sub‐domains on Faradaic processes at GO flakes is not known. In this work we carry out single‐entity electrochemical studies of N‐doped GO nanostructures using scanning electrochemical cell microscopy (SECCM)^[^ ^14^ ^]^ in hopping mode, as schematically shown in Figure 1c, to investigate electrochemical heterogeneity and intrinsic activity in the VO^2+^ oxidation process. We complement nanoelectrochemistry experiments with correlative Raman analysis and computational simulations to understand what factors control activity. Only few examples of single‐entity studies have been reported with vanadium species; notably, Kaliyaraj Selva Kumar and Compton^[^ ^15^ ^]^ demonstrated single‐entity characterization of VO_2_^+^/VO^2+^ at carbon nanotubes and Kroner et al.^[^ ^16^ ^]^ studied single carbon fibers. To the best of our knowledge, there are no reports of single‐entity studies at GO nanostructures, despite significant interest on these nanomaterials for VRFB.

## 2 Results and Discussion

**Figure 2:**
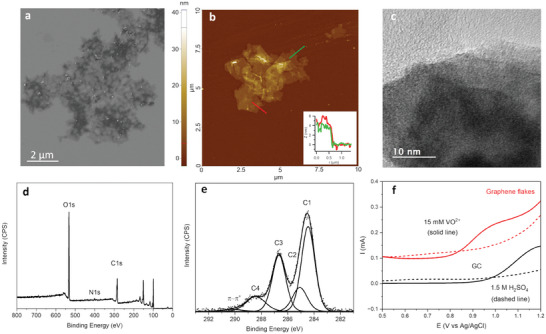
a) SEM image of drop‐cast GO. b) AFM height image of GO flakes; red and green lines indicate regions where height profiles were analyzed. c) TEM image of GO flakes. XPS d) survey and e) C1s spectrum and best‐fit. f) Voltammetry of GC and of N‐doped GO disk electrodes (geometric area = 0.196 cm^2^) in 15.0 mm VO^2+^ in 1.5 m H_2_SO_4_ at 50 mV s^−1^. Dashed lines indicate the response of the same electrodes in 1.5 m H_2_SO_4_ supporting electrolyte.

N‐doped GO nanostructures were obtained via electrochemical exfoliation of graphite in aqueous solutions containing ammonium salts (Figure S1, Supporting Information). **Figure** **2**a shows a scanning electron microscopy (SEM) image of drop‐cast N‐doped GO flakes, which display an irregular shape and are 8 ± 4 µm in size (see Figure S2, Supporting Information). Drop‐cast flakes display wrinkles and ripples as previously observed in the case of GO after exposure to water/humidity.^[^ ^17^ ^]^ Atomic force microscopy (AFM) images show step edge heights of 4–8 nm, Figure 2b and Figure S3 (Supporting Information), which are close to values reported for electrochemically exfoliated GO.^[^ ^18^ ^]^ This corresponds to nanostructures/stacks consisting of 2–10 layers, given that GO monolayers yield AFM heights of 0.5–1.7 nm depending on imaging conditions^[^ ^19^ ^]^ due to O‐functionalities and adsorbed water.^[^ ^17^, ^20^ ^]^ Transmission electron microscopy (TEM), Figure 2c, further supports the presence of few‐layer N‐doped GO, while also revealing residual nanoparticles (Figure S4, Supporting Information), likely resulting from the exfoliation process.

Chemical composition was characterized by XPS. Figure 2d shows the survey spectrum of N‐doped GO drop‐cast on Si; atomic compositions and best‐fit results are summarized in Table S2 (Supporting Information). The O/C was estimated at 65 at.% after correction for the oxygen contribution arising from the SiO_2_ support, in good agreement with other GO nanomaterials.^[^ ^21^ ^]^ The N 1s spectrum displays a peak at 401.9 eV (Figure S5, Supporting Information) assigned to protonated amine/pyridine functionalities^[^ ^22^ ^]^ and yielded N/C of 2.5 at.%. The high‐resolution C 1s spectrum, Figure 2e, was fitted using five contributions assigned to *sp^2^* (284.5 eV), *sp^3^* (285.1 eV), C─N/C─O functionalities (286.7 eV), ─COOH groups (288.4 eV) and π–π^*^ shake‐up (290.2 eV). The most prominent contributions arise from the graphitic scaffold (45%) and from C─N/C─O bonded carbons (29%), in agreement with the material consisting of graphitized and oxidized/nitrogenated regions. In summary, synthesis and deposition protocols were found to yield drop‐cast nanostructures consistent with N‐doped GO in terms of lateral size, thickness, and composition.^[^ ^21^ ^]^

The electrochemical response of exfoliated N‐doped GO materials was first investigated using drop‐cast disk electrodes in a conventional three‐electrode cell. Figure 2f shows linear sweep voltammograms (LSV) of a polished glassy carbon (GC) and of GC modified with electrochemically exfoliated N‐doped GO, in 1.5 m H_2_SO_5_ at 50 mV s^−1^ in the absence and presence of 15.0 mm of vanadyl sulfate. In supporting electrolyte, no anodic currents are observed until the onset of water oxidation. The capacitive background current was found to be higher for N‐doped GO than for polished GC electrodes, in agreement with the expected higher microscopic area and the presence of O‐ and N‐functionalities at these drop‐cast electrodes.^[^ ^7^, ^15^, ^23^ ^]^ In the presence of vanadyl cations, an anodic peak is observed at N‐doped GO electrodes, rising at ca. 0.8 V versus Ag/AgCl due to oxidation to pervanadyl species. A vanadyl/pervanadyl Faradaic peak is also observed at polished GC surfaces but at significantly larger overpotentials, in agreement with prior work showing sluggish vanadyl oxidation at graphitized electrodes that are devoid of heteroatom functionalities.^[^ ^6f^ ^]^

GO nanostructures were then investigated at the nanoscale via SECCM. Figure 1c illustrates the SECCM measurement configuration: a single‐barrelled nanopipette containing electrolyte and a quasi‐reference palladium hydride (PdH) electrode^[^ ^24^ ^]^ establishes electrochemical contact with the sample (working electrode) via a meniscus. The pipette is scanned in hopping mode to map the response of GO flakes drop‐cast onto a (N‐free) graphitized carbon thin‐film substrate deposited on a Si wafer. The thin‐film carbon substrate is topographically smooth and possesses a homogeneous electrochemical response, as previously reported;^[^ ^25^ ^]^ furthermore, it displays a sluggish response toward vanadyl oxidation,^[^ ^6f^ ^]^ thus offering good electrochemical contrast for studies of GO.

**Figure 3:**
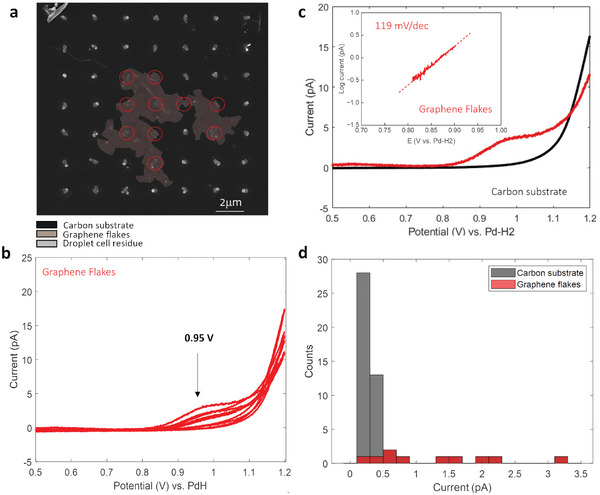
a) SEM image of a N‐doped GO flake on a carbon thin‐film substrate after probing via SECCM. The electrolyte residues are arranged in a visible grid pattern; points at which the GO flake was contacted are highlighted in red. b) LSV obtained at grid points contacting the GO flake (red circles in (a)), at 50 mV s^−1^ using 15.0 mm VO^2+^ in 0.150 m H_2_SO_4_. c) LSV at GO sub‐domain (red) compared to the average response at the thin‐film carbon substrate (black); the inset shows the average Tafel plot at the GO location. d) Histogram of current at 0.95 V versus PdH measured at points in the SECCM grid; the histogram for currents measured at GO and substrate locations are shown in red and grey, respectively.

Voltammetry experiments using SECCM consisted of probing drop‐cast samples at a series of locations in a square grid pattern. An LSV was obtained at each grid point at 50 mV s^−1^ using 15.0 mm VO^2+^ in 0.150 m H_2_SO_4_ as electrolyte under ambient conditions. Note that a lower concentration of H_2_SO_4_ was used compared to measurements at disk electrodes; this was necessary due to droplet instabilities in SECCM measurements observed at higher supporting electrolyte concentrations. **Figure** **3**a shows an SEM image of a GO flake after SECCM characterization with a 7 × 7 grid; the 49 probed points are evident thanks to the presence of electrolyte residue that is left at the surface at each contact location. At 10 points in the grid (red circles) the SECCM probe established contact with a sub‐domain of the GO flake, while the remainder 39 points contacted the carbon thin‐film substrate. Figure 3b shows LSV at GO‐contacted points (see also Figures S6 and S7, Supporting Information): the majority yielded an anodic peak or a clear increase in anodic current ≈0.8–0.9 V versus PdH. In contrast, none of the points at which the carbon thin‐film substrate was contacted yielded significant currents or anodic peaks in this potential region (Figure S6, Supporting Information). At the thin‐film carbon substrate, vanadyl oxidation currents increase at a more anodic potential, in agreement with previous studies of the thin‐film support that show that vanadyl oxidation currents reach a maximum at more anodic potentials than 1.2 V versus PdH under similar conditions (see also computational results discussed below).^[^ ^6f^ ^]^ This contrast is clearly illustrated in Figure 3c, which compares the LSV at the GO sub‐domain with the highest current at 0.95 V versus the LSV at the carbon substrate: a well‐resolved anodic current and a featureless response are visible at the N‐doped GO and the graphitic carbon substrate, respectively. The Tafel plot calculated at this GO location shows good linearity with a slope of 119 mV, which suggests that the current is controlled by an electrochemical rate‐determining step.

The LSV data reveals a high degree of electrochemical heterogeneity across sub‐domains of a GO nanostructure; this is evident from a spread in current values over the 0.8–1.0 V potential window. Figure 3d shows a histogram of currents at 0.95 V for GO (red) and substrate (grey) contacting points. While the response of the substrate is narrowly dispersed at low current values, the GO domains show widely spread current intensities ranging over 0–3.2 pA. Furthermore, several points (40%) yield currents at 0.95 V that are indistinguishable from those of the inert carbon substrate. Finally, several LSV were found to display a sigmoidal waveform consistent with that arising from radial diffusion at a microelectrode, as shown in Figure 3c. This suggests that the current is likely dominated by a highly active region with significantly faster kinetics relative to that of the carbon substrate and with a lateral size that is much smaller than the diameter of the nanopipette.

**Figure 4:**
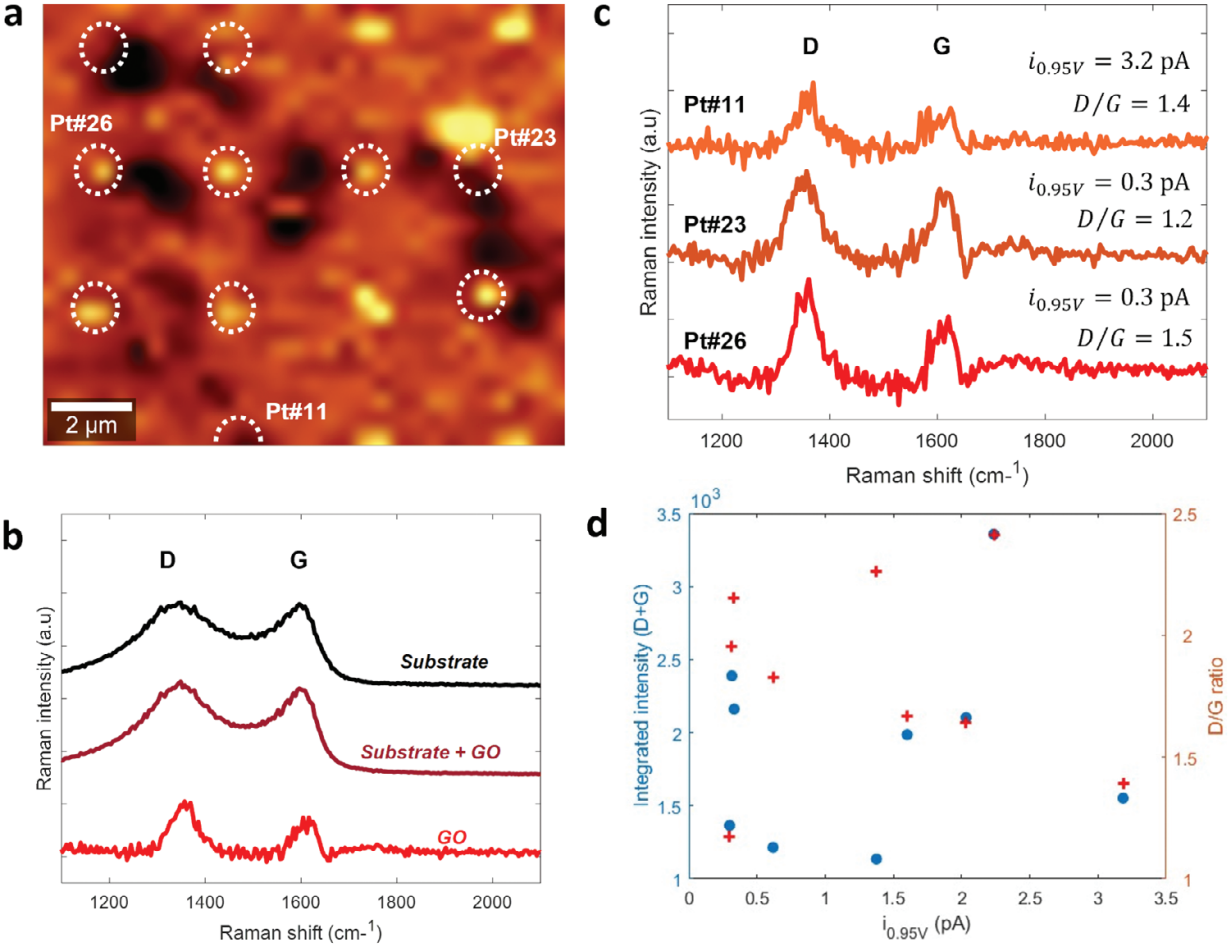
a) Raman intensity map at 524 cm^−1^ rel. shift showing electrolyte residues and the outline of the GO flake; indexed points (Pt#) correspond to those whose Raman spectra is shown in panel (c) (see also Figure S6, Supporting Information). b) Average Raman spectra at locations corresponding to the substrate (black) and to the GO flake onto the substrate (brown); the difference spectrum (red) shows contributions from the N‐doped GO flake alone. c) Raman of N‐doped GO at three different points in the SECCM grid; the corresponding current at 0.95 V versus PdH and D/G ratio is listed above each trace. d) Scatter plots of D/G ratios and integrated Raman intensity versus anodic currents at 0.95 V.

Correlative Raman‐electrochemical studies by other groups using outer‐sphere redox couples, such as ferrocenium/ferrocene, have previously revealed strong correlations between carbon spectral parameters that depend on defects and charge transfer rate constants.^[^ ^11d^ ^]^ To investigate whether similar correlations can be observed using vanadyl species, we carried out Raman mapping of the GO flake in Figure 3. The Raman map at 524 cm^−1^, in **Figure** **4**a, shows intensity contrast due to the Si wafer support^[^ ^26^ ^]^ and V(V) oxides,^[^ ^27^ ^]^ thus enabling accurate localization of the electrolyte residues (circles). The grid was also evident at the same positions at the energy corresponding to the Rayleigh line, as shown in Figure S8 (Supporting Information). The outline of the GO flake can also be seen in Figure 4a thanks to the attenuation of the Si signal from the support wafer. This allows analysis in the 1200–1800 cm^−1^ region diagnostic for carbon materials^[^ ^28^ ^]^ at the exact locations that had been probed electrochemically. Figure 4b shows the average spectrum of the 10 points at which the GO flake had been probed via SECCM (brown), that of the carbon thin‐film substrate (black), and the resulting difference spectrum (red) displaying the GO flake contribution. The spectrum shows two peaks assigned to the characteristic D (ca. 1350 cm^−1^) and G (ca. 1600 cm^−1^) bands of graphenes. The G band is assigned to E_2g_ stretching modes of *sp^2^‐*carbon and the D band to the A_1g_ breathing mode of aromatic rings, which becomes Raman‐active due to structural disorder.^[^ ^22^, ^28^, ^29^ ^]^ D/G height and area ratios are diagnostic for the density of defects in graphene and were found to be 1.5 and 2.1, respectively, in agreement with typical values for GO nanomaterials.^[^ ^21^, ^30^ ^]^ The full width at half maximum of G and D bands are 51 and 72 cm^−1^, respectively, and suggest that the flake is best described within the high‐defect‐density regime of Raman spectral parameters.^[^ ^31^ ^]^ Based on work by Childres et al.^[^ ^30^ ^]^ on oxidized graphene, an average defect‐defect separation of 3.6 nm was estimated from the area ratio.

Difference spectra were also calculated for each of the ten individual points in the SECCM grid that contacted the GO (Figure S8, Supporting Information), and best fits of individual difference spectra were obtained for all but one of the ten points probed via SECCM (Table S3, Supporting Information). Figure 4c shows examples of spectra at three GO locations that displayed various anodic currents. No trends relating Raman spectral parameters to the electrochemical performance are evident, as confirmed also by scatter plots of D/G ratios and integrated peak intensities versus the LSV current at 0.95 V, in Figure 4d. Assuming the GO structure to be unchanged by the electrochemical probing, this result indicates that the density of structural defects is not likely to be a factor in determining vanadyl oxidation activity. Defects that contribute to the final carbon Raman spectrum in N‐doped GO encompass edges, vacancies, sp^3^‐centers in the carbon scaffold, and O‐/N‐functionalities.^[^ ^7^, ^9^, ^32^, ^33^ ^]^ The absence of correlation between electrochemical performance and D/G parameters, which depend on defect density but are typically insensitive to the nature of defects,^[^ ^31b^ ^]^ suggests that the chemical nature of defects might be more important to explain the origin of vanadyl activity at GO sub‐domains than their density. Interestingly, this is in contrast with prior work on correlative Raman‐electrochemical studies of outer‐sphere redox couples such as ferrocenium/ferrocene,^[^ ^11d^ ^]^ but is consistent with current evidence on the role of specific interactions in the electrochemical response of vanadium species. Several groups have reported that chemisorption of vanadium species takes place at oxidized functionalities and plays a role in determining activity.^[^ ^34^ ^]^ Also, it is consistent with findings from our group that support the role of basic N‐functionalities in improving vanadyl charge transfer rates and reversibility.^[^ ^6f^ ^]^ Therefore, we hypothesize that specific surface functionalities that change the local *chemistry* of GO sub‐domains give rise to activity and are at the origin of electrochemical heterogeneity.

LSV data reveal that GO flake nanostructures consist of sub‐domains with widely varying degrees of activity toward vanadyl oxidation. Several of the points probed at GO locations via SECCM gave a response that is indistinguishable from that of the substrate; therefore, it is possible to conclude that the area of catalytically “silent” domains in GO flakes can be as large as the SECCM contact areas, i.e., ca. 1.5 µm2 (see Figure S6, Supporting Information). The relevant size of some of the active domains within GO flakes, on the other hand, might be first approximated by examining the LSV for the most active point in Figure 3b. This LSV displays a nearly sigmoidal waveform characteristic of microelectrode voltammograms, with a plateau at 3.2 pA akin to that of a diffusion‐limited current (*i_d_*). This suggests the presence of an active region with a small area, relative to that of the droplet cell, that is responsible for the observed anodic current. Neglecting mass transport limitations in the nanopipette probe, the radius of a single equivalent circular sub‐domain (*r*) in the GO flake that would give rise to this can be estimated based on:^[^ ^35^ ^]^

(1) $$i_{d}=4nrFDc$$

where *F* is Faraday's constant, *n* is the number of electrons, *D* is the vanadyl diffusion coefficient (2.1 × 10^−6^ cm^2^ s^−1^)^[^ ^6f^ ^]^ and *c* is the vanadyl concentration. For *i_d_* = 3.2 pA the active sub‐domain radius calculated from Equation (1) is 2.6 nm, i.e., much smaller than the contact radius of the nanopipette probe (ca. 0.7 µm) and smaller than the average inter‐defect distance determined from the Raman map.

**Figure 5:**
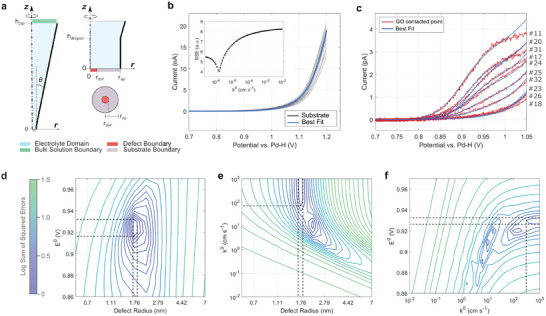
a) Model implemented in finite element simulations. b) Best‐fit (blue) of the LSV of the carbon thin‐film substrate (black); inset shows SSE plots near the minimum. c) Best‐fits (blue) of the ten LSV measured at GO points (black). d–f) Example of contour plots of the SSE near the best‐fit minimum for point #20; lines show +30% increases in SSE relative to the preceding contour line. Dashed lines indicate the 30% uniqueness range of best‐fit parameter determinations along each axis direction.^[^ ^6^, ^37^ ^]^

A more in‐depth analysis of the anodic response at GO sub‐domains was carried out by simulating LSV curves using finite element methods. This enables us to account for mass‐transport effects in the nanopipette probe, to expand the analysis to all experimental LSV curves, and to obtain quantitative information on charge transfer kinetics. A model of the pipette probe was implemented on COMSOL Multiphysics, as shown in the scheme in **Figure** **5**a; details of geometry and dimensions are described in Text S1 (Supporting Information). The probe aperture was fixed at a radius equal to that of electrolyte residues (0.7 µm), while the height of the meniscus was assumed to be equal to the probe radius, as in previous work.^[^ ^36^ ^]^ Vanadyl and pervanadyl concentrations were set to 15 and 0 mm, respectively, at time zero and at the bulk solution boundary, and diffusion coefficients were fixed at literature values.^[^ ^6f^ ^]^

The anodic wave was simulated as an electrochemical step (E‐step) involving a 1‐electron transfer according to Butler–Volmer kinetics (Text S1, Supporting Information) assuming charge transfer coefficient α  = 0.5.^[^ ^6^, ^38^ ^]^ A first test of the model consisted in simulating the response of the thin‐film carbon substrate for the points in Figure 3a shown to be in contact with the support electrode exclusively. The LSV at points #1‐#3 of the grid were excluded from analysis because of droplet size variability during the first probe approaches in a grid (see Figure S6, Supporting Information). LSV was simulated over a range of heterogeneous rate constant ($k_{sub}^{0}$) values (see Table S6, Supporting Information) with formal potential fixed as in our previous work^[^ ^6f^ ^]^ after applying a correction of +67 mV to account for the differences in the reference electrode and in acid concentration. The sum of square errors (SSE) calculated over all LSV curves in the dataset was then used as a figure of merit to identify a best fit. Figure 5b shows the best fit (blue) obtained for $k_{sub}^{0}=$ 1.6 × 10^−6^ cm s^−1^, and the dataset of 36 LSV (black) recorded at the carbon thin‐film substrate; the SSE versus $k_{sub}^{0}$ plot in the inset displays a well‐defined minimum at the above value. The rate constant of the substrate compares very well with a value of 9.34 × 10^−7^ cm s^−1^ determined from cyclic voltammograms at macroscopic disk electrodes of the same thin‐film carbon material.^[^ ^6f^ ^]^ This result supported that the model geometry, in particular the assumed values of probe radius and meniscus height which are known to affect simulated currents,^[^ ^39^ ^]^ are consistent with and result in a satisfactory description of the response at SECCM grid points.

To simulate the response at the GO sub‐domains, the contacted surface was modeled as a disk with a highly active area of radius *r_def_* surrounded by an electrode surface with heterogeneous rate constant $k_{sub}^{0}$ matching the best‐fit for the carbon substrate. This approach is based on our prior work on the simulation of a defect response on 2D materials^[^ ^35a^ ^]^ and assumes that outside the GO active domain, the electrochemical response is controlled by the substrate. LSV curves were simulated by varying *r_def_*, the heterogeneous rate constant ($k_{def}^{0}$) and the formal potential ($E_{def}^{0^{\prime }}$) at the defect site (see Table S6, Supporting Information). Figure 5c shows the best fit obtained for each of the 10 LSV in the region 0.7–1.05 V versus PdH; it is evident from this comparison that the model satisfactorily captures the main trends across the dataset.

**Table** **1** reports best‐fit parameters for each of the ten points; current values for each of the LSV are also listed to facilitate comparisons. Three of the points were found to possess the lowest rate constants and smallest active areas; they correspond to LSV that are challenging to discriminate from those of the substrate and that resulted in SSE surfaces with poorly defined minima (Figure S9, Supporting Information). The remaining seven points yielded heterogeneous rate constants >10 cm s^−1^ and their defect radii were found to correlate strongly with anodic currents. Best‐fit minima for these seven curves were well‐defined in the ($E_{def}^{0^{\prime }}$, *r_def_*) plane, as illustrated in Figure 5d for, e.g., #20 in the grid sequence (see also Figure S9, Supporting Information); however, the error surface yields shallow minima along the $k_{def}^{0}$ axis (Figure 5e,f). This is also clear from an examination of fit uncertainties, obtained by defining the range of $k_{def}^{0}$ values that results in a 30% increase in the SSE relative to the best‐fit minimum^[^ ^6^, ^37^ ^]^ (see Supporting Information). Uncertainty ranges are generally large around the $k_{def}^{0},$ reflecting the shallowness of best‐fit minima. Nonetheless, the lower end of each range can be regarded as a lower boundary estimate for a rate constant describing the kinetics of vanadyl oxidation.

**Table 1:** Kinetic rate constants and formal potentials obtained from simulations and best‐fits; potentials are reported versus PdH. The anodic currents recorded at the same points are also shown for comparison; points are indexed according to their position in the grid, as shown in Figure S6 (Supporting Information). Uncertainties on $k_{def}^{0}$ were established by identifying the widest uniqueness range^[^ ^6^, ^37^ ^]^ yielding 30% increase in the SSE relative to the best‐fit minimum.

GO grid point	$k_{def}^{0}$ [cm s^−1^]	$E_{def}^{0^{\prime }}$ [V]	*r*_*def*_ [nm]	Current @0.95 V [pA]
#11	2.5 × 10^1^ (8.1;^b)^1.0 × 10^3^)	0.90	2.79	3.2
#17	1.0 × 10^2^ (4.1 × 10^1^;^b)^1.0 × 10^3^)	0.92	1.40	1.6
#18^a)^	1.0 × 10^−2^ (^b)^1.0 × 10^−2^; 1.6 × 10^−1^)	0.97	0.56	0.3
#20	1.0 × 10^3^ (7.2 × 10^1^;^b)^1.0 × 10^3^)	0.93	1.76	2.0
#23^a)^	4.0 × 10^−1^ (^b)^1.0 × 10^−2^; 1.0)	0.89	0.56	0.3
#24	4.0 × 10^2^ (7.8 × 10^1^;^b)^1.0 × 10^3^)	0.92	1.11	1.4
#25	1.6 × 10^2^ (4.3 × 10^1^; 4.4 × 10^2^)	0.92	0.70	0.8
#26^a)^	4.0 × 10^−2^ (^b)^1.0 × 10^−2^; 6.0 × 10^−2^)	0.87	0.56	0.3
#31	1.0 × 10^3^ (4.4 × 10^1^;^b)^1.0 × 10^3^)	0.92	1.76	2.2
#32	1.6 × 10^1^ (6.5; 4.0 × 10^1^)	0.89	0.56	0.6

^a)^ Grid points that did not yield well‐defined minima;

^b)^ Uncertainty range is at the limit of the parametric sweep of the simulation, as specified in Table S6 (Supporting Information).

Once the uncertainty range in $k_{def}^{0}$ in Table 1 is considered for all points, all probed locations that result in activity enhancements relative to the substrate are observed to require rate constants greater than 1–10 cm s^−1^ to satisfactorily model the LSV response. Such values are comparable or slightly lower than those reported for fast heterogeneous electron‐transfer reactions at nanofabricated electrodes.^[^ ^40^ ^]^ Importantly, they suggest that active sub‐domains in N‐doped GO can indeed display the very fast kinetics needed to meet the proposed thresholds for the development of highly active VRFB electrode materials. Values of *k* ^0^ at these active sites^[^ ^40^ ^]^ are larger than the ca. 10^−3^ cm s^−1^ reported for individual bamboo multiwalled carbon nanotubes^[^ ^15^ ^]^ and single carbon fibers,^[^ ^16^ ^]^ suggesting that sub‐domains at carbon nanomaterials can be potentially engineered to display very fast vanadyl oxidation kinetics. Finally, it is interesting to note that $E_{def}^{0^{\prime }}$ values are approximately constant at ca. 0.92 V versus PdH, a value more anodic than $E_{sub}^{0^{\prime }}$. We speculate this to arise from binding interactions that stabilize vanadyl cations at the electrode surface relative to the pervanadyl species.^[^ ^41^ ^]^ A relatively modest extra‐stabilization of ca. 5 kJ mol^−1^ is sufficient to explain a +50 mV shift and is consistent with experimental evidence of vanadyl binding at O‐functionalities reported by several groups.^[^ ^34^ ^]^

It is important to note that LSV were satisfactorily simulated by assuming the current to arise from a single axisymmetric disk‐shaped defect, however, this is likely a significant simplification of what is effectively a very complex N‐doped GO surface. First, similar currents and waveforms could be equally expected to arise from a collection of individual isolated active sites with *r_def_* smaller than those reported in Table 1 and located off‐axis within the perimeter of the droplet cell. For instance, considering vanadyl oxidation to occur at isolated functionalities with their radii limited by that of a bound vanadyl aquo complex (0.2 nm),^[^ ^42^ ^]^ the presence of 14 well‐separated active functionalities within the 1.5 µm^2^ area of the droplet each contributing an *i_d_* as in Equation (1), could also satisfactorily result in a 3.2 pA current plateau (as for point #11 in the grid). Given C_COOH_/C_tot_ and N/C_tot_ of ca. 10 and 2.5 at.%, respectively, obtained from XPS (Table S2, Supporting Information) and a carbon areal density in graphene of 3.8 × 10^7^ atoms µm^−2^ we can conclude that only a small fraction of functionalities is responsible for observed activity.

Second, imposing a central axis of symmetry and an area surrounding the defect whose response is characteristic of the carbon thin‐film substrate does not necessarily result in satisfactory or realistic solutions for other GO flakes characterized via SECCM. For instance, Figure S10 (Supporting Information) shows the results of a SECCM experiment and the corresponding SEM image with grid points classified based on the location contacted (GO vs substrate). The LSV response of the carbon thin‐film substrate was successfully simulated using the same parameters as for the substrate in Figure 5b, except for a probe aperture *r_ap_* =  1.2 µm that reflects the use of a different nanopipette probe. The LSV at GO locations show sigmoidal waveforms with similar trends as those in Figure 3c; however, the current plateau is observed at ca. 80 pA which would require a model with a defect radius of ca. 65 nm. This value is greater than 5% of the probe aperture radius and it appears unlikely that active regions/sites would concentrate at the center of the droplet cell, thus maintaining axial symmetry. Importantly, the LSV currents of substrate‐ and GO‐contacted points were not observed to converge at the anodic limit; this suggests that diffusion fronts from these two types of surfaces are sufficiently distant from each other to result in additive current contributions within the relatively small 1.2 µm radius of the droplet cell. Therefore, the generality of the axisymmetric model implemented herein is likely limited and a more realistic model might be needed to simulate these larger currents. Allowing for diffusion to line/band defects, e.g., from step edges at the GO flake, or to 3D defects/features, e.g. due to GO roughness or fragments, would result in higher plateau currents than those obtained from radial diffusion;^[^ ^35^, ^43^ ^]^ both types of defects appear reasonable given the irregular nature of GO flakes. Nonetheless, in the case of the simulated voltammetry in Figure 5, both the magnitude of faradaic charges and the height information obtained from nanopipette positioning (Text S2, Supporting Information) are consistent with the adoption of a disk geometry. Therefore, despite the limitations of the model implemented, our findings show that certain defects and active sub‐domains can display extremely fast rates, offering opportunities to further optimize the response of GO‐based carbon electrodes in VRFB applications.

Finally, the use of Butler‐Volmer kinetics to simulate the flux at the electrode surface possibly constitutes a simplification over a choice of more complex approaches such as the Marcus‐Hush formalism. However, work by Felberg^[^ ^44^ ^]^ shows that the responses predicted by Marcus–Hush and Butler–Volmer tend to converge for fast heterogeneous charge transfer. Based on Feldberg's analysis, at room temperature, assuming *r =* 2 nm and a reorganization energy of ≈1 eV, the maximum value of *k^0^* for vanadyl species that would lead to meaningful differences between the two formalisms is ≈10^−3^ cm s^−1^. This is much lower than any of the lower boundary estimates in Table 1 thus supporting that the choice of the Butler–Volmer formalism is not likely to significantly impact the conclusions.

## 3 Conclusion

N‐doped GO was synthesized via electrochemical exfoliation and characterized using a combination of methods. The electrochemical response of sub‐domains within an isolated GO flake on electrochemically inert carbon thin‐film substrates was characterized via SECCM^[^ ^14b^ ^]^ by mapping the electrochemical response using VO^2+^‐containing electrolyte. Correlative Raman‐SECCM mapping was also performed to investigate any possible relationship between the defect density and activity toward vanadyl oxidation. Results show that even an individual N‐doped GO flake can display significant electrochemical heterogeneity: small defects/active sites appear to be interspersed between relatively large sub‐domains that are inert toward vanadyl oxidation. Correlative Raman‐SECCM analysis suggests that the defect density is not a good predictor of electrochemical activity but that the specific chemistry of local functionalities might be a more important factor for understanding vanadyl oxidation rates at such sites. We support our analysis with finite element simulations of the electrochemical response that strongly suggest that active sub‐domains in the GO flake are smaller than the mean inter‐defect distance estimated from Raman spectra. Despite possessing radii smaller than 3 nm, these sub‐domains can display very fast heterogeneous rate constants (>1 cm s^−1^) that account for the activity observed. Simulations also support that binding interactions at these highly localized functionalities could be at play and facilitate fast electron transfer.

It is not possible on the basis of our results alone to identify the exact type of functionalities responsible for high activity, however, our findings provide a new platform for the assessment of the intrinsic properties of these nanomaterials toward VRFB applications. Importantly, they suggest that N‐/O‐functionalized graphene‐based electrodes hold great promise for VRFB applications: active sub‐domains can indeed display the very high intrinsic activities that have been proposed as a requirement for the fabrication of competitive devices. Given that the graphitic scaffold motif is common to a wide range of nanocarbons we also speculate that it is possible to engineer similar sub‐domains on other carbon nano‐entities such as nanotubes, nanohorns, carbon dots, etc. Therefore, careful control over functionalities and engineering of nanocarbon assemblies holds strong potential to deliver high‐performing 3D electrode architectures for VRFB technologies.

## 4 Experimental Section

### Materials

Vanadyl sulfate hydrate (99.999%), sulfuric acid (99.99%), ammonium sulfate (99%), methanol (HPLC, 99.9%), ethanol (HPLC, 99.8%) and Nafion 117 (ca. 5% in alcohols/water), were purchased from Merck/Sigma Aldrich and used as received. Wafers (300 nm thermally grown oxide on Si) and GC (5 mm o.d. Sigradur HTW) were used as substrates. Graphite targets were obtained from Kurt J. Lesker (99.999%); alumina slurries and polishing cloths were purchased from Buehler.

### Materials Synthesis and Electrode Fabrication

Graphitized carbon thin film electrodes for nanoelectrochemistry studies were fabricated on SiO_2_/Si wafers using published protocols.^[^ ^25^, ^45^ ^]^ Briefly, films were first deposited in a DC‐magnetron sputtering chamber from a graphite target at 1–2 × 10^−2^ mbar in Ar. Deposited carbon films were annealed at 900 °C under N_2_ flow for 1 h to yield graphitized thin‐film electrodes of thickness of 83 ± 1 nm.^[^ ^25b^ ^]^ Graphene flakes were prepared via electrochemical exfoliation as previously reported^[^ ^22d^ ^]^ using graphite targets as precursor materials (see Figure S1, Supporting Information). The targets were first cleaned by sonication in methanol (20 min), then immersed in a 0.1 m (NH_4_)_2_SO_4_ solution and connected to a DC power supply (Agilent U8001A). Electrochemical exfoliation was carried out at 10 V for a min of 5 h. The solution was left to settle overnight; then, the supernatant was collected in water (18.2 MΩ cm) via centrifugation (6000 rpm for 1 h). The sediment was then dispersed in deionized water several times until the aqueous phase reached pH 6. Graphene flakes were then redispersed to obtain a stock dispersion (55.4 mg mL^−1^); the yield was calculated based on the freeze‐dried weight of the graphene flakes.

### Characterization

XPS was performed on an Omicron ESCA system with a monochromatic Al Kα source (1486.7 eV). Survey scans were obtained at 50 eV pass energy, while high‐resolution spectra were obtained at 15 eV pass energy. All spectra were corrected using a Shirley background and best fitted with Voigt functions (CasaXPS). Atomic compositions were determined from peak areas, after correction for relative sensitivity factors. SEM (Zeiss Ultra FE) was carried out using secondary electron and Lens detectors at acceleration voltage of 5 kV. TEM (JEM 2100 HR, Jeol) was carried out at 200 kV with STEM and EFTEM resolutions of 1 nm. AFM (Park system NX10) images were obtained in non‐contact mode using PPP‐NCHR probes with nominal 42 N/m force constant and 350 kHz resonant frequency. Raman spectroscopy/microscopy (Witec alpha 300R) was performed using a confocal scanning system at 532 nm excitation; data analysis was carried out using Witec Project 5.1 and data processing software (Matlab, Mathematica). Raman best fits were carried out with commercial software (CasaXPS) assuming Voigt peak shapes.

### Electrochemical Studies

Electrochemical measurements using disk electrodes were carried out on a potentiostat (Autolab Metrohm or Pine) in a three‐electrode cell with a graphite rod as counter electrode and Ag/AgCl as reference electrode. Bare GC disks or GC disks modified with graphene nanostructures were used as working electrodes. Disks were polished with alumina slurries to a mirror finish, as previously reported.^[^ ^6f^ ^]^ Modified GC disks were prepared via drop casting onto the polished GC surface to yield 0.25 mg cm^−2^ loadings. Inks were prepared using 135 µL of ethanol, 25 µL of Nafion, and 340 µL of deionized water containing 5.0 mg of graphene flakes, followed by vortex agitation (1 min). All disk voltammetry was carried at 50 mV s^−1^ in 1.5 m H_2_SO_4_ with/without 15.0 mM VO^2+^, as noted in each case. Prior to all measurements the cell was thermostated at 20 °C and the electrolyte was purged 20 min with Ar.

Nanoscale electrochemical measurements were performed on a Park NX10 instrument (Park Systems) using the SmartScan software, in a Faraday cage and at ambient conditions. Samples were prepared by drop‐casting 2 µL of a 5 µg mL^−1^ stock graphene flake dispersion onto the graphitized carbon thin‐film substrates; samples were dried in air for 1 h prior to measurement. A single‐barrelled nanopipette was used as probe, fabricated as detailed elsewhere;^[^ ^25a^ ^]^ nanopipettes were filled with 15.0 mm VOSO_4_ in 0.150 m H_2_SO_4_ electrolyte using a pipette filler (MicroFil MF34G‐5, World Precision Instruments, USA). The quasi reference counter electrode (QRCE) was a PdH wire (0.25 mm o.d., 5 cm, Goodfellow UK) fabricated electrochemically.^[^ ^14^, ^25^, ^46^ ^]^ The QRCE was inserted at the top end of the filled nanopipette probe. Probes were approached to the sample at 1.0 µm s^−1^ with threshold currents set at 3.0 pA and were then scanned in hopping mode following a raster pattern. Linear sweep voltammograms from +0.4 to 1.2 V versus PdH at 0.050 V s^−1^ were obtained at each point in the grid; currents are shown after correction by the plateau preceding the Faradaic potential region. Electrochemical simulations were carried out using COMSOL Multiphysics v.5.4 and details of the geometry, parameters, boundary conditions, and parametric sweeps are reported in Text S1 (Supporting Information).

## Conflict of Interest

Kim McKelvey reports that the equipment used for some of the reported experiments was provided by Park Systems Corp.

## Supporting information



Supporting Information

## Data Availability

The data that support the findings of this study are available from the corresponding author upon reasonable request.

## References

[1] M. Skyllas‐Kazacos , C. Menictas , Encyclopedia of Energy Storage (Ed.: L. F. Cabeza ), Elsevier, Oxford 2022, pp. 407–422.

[2] M. Skyllas‐Kazacos , M. H. Chakrabarti , S. A. Hajimolana , F. S. Mjalli , M. Saleem , J. Electrochem. Soc. 2011, 158, R55.

[3] a) K. J. Kim , M.‐S. Park , Y.‐J. Kim , J. H. Kim , S. X. Dou , M. Skyllas‐Kazacos , J. Mater. Chem. A 2015, 3, 16913;

[4] a) J. Noack , N. Roznyatovskaya , J. Kunzendorf , M. Skyllas‐Kazacos , C. Menictas , J. Tübke , J. Energy Chem. 2018, 27, 1341;

[5] a) Q. Ma , X.‐X. Zeng , C. Zhou , Q. Deng , P.‐F. Wang , T.‐T. Zuo , X.‐D. Zhang , Y.‐X. Yin , X. Wu , L.‐Y. Chai , Y.‐G. Guo , ACS Appl. Mater. Interfaces 2018, 10, 22381; 29902919 10.1021/acsami.8b04846

[6] a) Y. Huang , Q. Deng , X. Wu , S. Wang , Int. J. Hydrog. Energy 2017, 42, 7177;

[7] a) M. Liu , Z. Xiang , J. Piao , J. Shi , Z. Liang , Electrochim. Acta 2018, 259, 687;

[8] a) S. M. Taylor , A. Pătru , D. Streich , M. El Kazzi , E. Fabbri , T. J. Schmidt , Carbon 2016, 109, 472;

[9] a) X. Michel Myures , S. Suresh , J. Energy Storage 2023, 58, 106387;

[10] a) P. Han , H. Wang , Z. Liu , X. Chen , W. Ma , J. Yao , Y. Zhu , G. Cui , Carbon 2011, 49, 693;

[11] a) A. G. Güell , N. Ebejer , M. E. Snowden , J. V. Macpherson , P. R. Unwin , J. Am. Chem. Soc. 2012, 134, 7258; 22486239 10.1021/ja3014902

[12] a) P. Saha , M. M. Rahman , C. M. Hill , Electrochem. Sci. Adv. 2022, 2, 2100120;

[13] a) H. Lee , N. Son , H. Y. Jeong , T. G. Kim , G. S. Bang , J. Y. Kim , G. W. Shim , K. C. Goddeti , J. H. Kim , N. Kim , H.‐J. Shin , W. Kim , S. Kim , S.‐Y. Choi , J. Y. Park , Nanoscale 2016, 8, 4063; 26819189 10.1039/c5nr06469d

[14] a) C. L. Bentley , M. Kang , P. R. Unwin , J. Am. Chem. Soc. 2019, 141, 2179; 30485739 10.1021/jacs.8b09828

[15] A. Kaliyaraj Selva Kumar , R. G. Compton , ACS Catal. 2022, 12, 4754.

[16] I. Kroner , M. Becker , T. Turek , ChemElectroChem 2020, 7, 4314.

[17] a) X. Su , R. K. Pandey , J. Ma , W. C. Lim , C. K. Ao , C. Liu , H. Nakanishi , S. Soh , Soft Matter 2022, 18, 3546; 35445678 10.1039/d1sm01834e

[18] a) W.‐W. Liu , A. Aziz , ACS Omega 2022, 7, 33719; 36188239 10.1021/acsomega.2c04099 PMC9520741

[19] a) C. J. Shearer , A. D. Slattery , A. J. Stapleton , J. G. Shapter , C. T. Gibson , Nanotechnology 2016, 27, 125704; 26894444 10.1088/0957-4484/27/12/125704

[20] a) Q. Tan , Y. Fan , Z. Song , J. Chen , L. Chen , J. Mol. Model. 2022, 28, 57; 35137256 10.1007/s00894-022-05045-7

[21] K. Z. Donato , H. L. Tan , V. S. Marangoni , M. V. S. Martins , P. R. Ng , M. C. F. Costa , P. Jain , S. J. Lee , G. K. W. Koon , R. K. Donato , A. H. Castro Neto , Sci. Rep. 2023, 13, 6064. 37055491 10.1038/s41598-023-33350-5 PMC10102077

[22] a) A. Artemenko , A. Shchukarev , P. Štenclová , T. Wågberg , J. Segervald , X. Jia , A. Kromka , IOP Conf. Ser. Mater. Sci. Eng. 2021, 1050, 012001;

[23] a) F. Béguin , K. Szostak , G. Lota , E. Frackowiak , Adv. Mater. 2005, 17, 2380;

[24] M. J. Vasile , C. G. Enke , J. Electrochem. Soc. 1965, 112, 865.

[25] a) M. Brunet Cabré , D. Spurling , P. Martinuz , M. Longhi , C. Schröder , H. Nolan , V. Nicolosi , P. E. Colavita , K. McKelvey , Nat. Commun. 2023, 14, 374; 36690615 10.1038/s41467-023-35950-1 PMC9870982

[26] T. S. Perova , R. A. Moore , K. Lyutovich , M. Oehme , E. Kasper , Thin Solid Films 2008, 517, 265.

[27] H. Radinger , F. Bauer , F. Scheiba , Batter. Supercaps 2023, 6, 202200440.

[28] a) A. C. Ferrari , D. M. Basko , Nat. Nanotechnol. 2013, 8, 235; 23552117 10.1038/nnano.2013.46

[29] S. Reich , C. Thomsen , Philos. Trans. R. Soc. London, A 2004, 362, 2271. 10.1098/rsta.2004.1454 15482979

[30] I. Childres , L. A. Jauregui , J. Tian , Y. P. Chen , New J. Phys. 2011, 13, 025008.

[31] a) I. Childres , L. Jauregui , W. Park , H. Caoa , Y. P. Chena , New Developments in Photon and Materials Research, (Ed.: J. I. Jang ), Nova Science Publishers, UK 2013 pp. 403;

[32] P. M. Nia , E. Abouzari‐Lotf , P. M. Woi , Y. Alias , T. M. Ting , A. Ahmad , N. W. Che Jusoh , Electrochim. Acta 2019, 297, 31.

[33] W. Li , J. Liu , C. Yan , Carbon 2013, 55, 313.

[34] a) J. Maruyama , T. Shinagawa , A. Hayashida , Y. Matsuo , H. Nishihara , T. Kyotani , ChemElectroChem 2016, 3, 650;

[35] a) M. B. Cabré , A. E. Paiva , M. Velický , P. E. Colavita , K. McKelvey , J. Phys. Chem. C 2022, 126, 11636;

[36] M. Brunet Cabré , A. E. Paiva , M. Velický , P. E. Colavita , K. McKelvey , Electrochim. Acta 2021, 393, 139027.

[37] a) B. Singh , A. Diwan , V. Jain , A. Herrera‐Gomez , J. Terry , M. R. Linford , Appl. Surf. Sci. 2016, 387, 155;

[38] D. Li , C. Batchelor‐McAuley , R. G. Compton , Appl. Mater. Today 2020, 18, 100404.

[39] M. E. Snowden , A. G. Güell , S. C. S. Lai , K. McKelvey , N. Ebejer , M. A. O'Connell , A. W. Colburn , P. R. Unwin , Anal. Chem. 2012, 84, 2483. 22279955 10.1021/ac203195h

[40] a) P. Sun , M. V. Mirkin , Anal. Chem. 2006, 78, 6526; 16970330 10.1021/ac060924q

[41] B. B. Blizanac , P. N. Ross , N. M. Markovic , Electrochim. Acta 2007, 52, 2264.

[42] J. Krakowiak , D. Lundberg , I. Persson , Inorg. Chem. 2012, 51, 9598. 22950803 10.1021/ic300202f PMC3490104

[43] a) H. Le , R. G. Compton , J. Electroanal. Chem. 2020, 866, 114149;

[44] S. W. Feldberg , Anal. Chem. 2010, 82, 5176. 20496865 10.1021/ac1004162

[45] J. A. Behan , E. Mates‐Torres , S. N. Stamatin , C. Domínguez , A. Iannaci , K. Fleischer , M. K. Hoque , T. S. Perova , M. García‐Melchor , P. E. Colavita , Small 2019, 15, 1902081. 10.1002/smll.201902081 31210002

[46] E. Daviddi , K. L. Gonos , A. W. Colburn , C. L. Bentley , P. R. Unwin , Anal. Chem. 2019, 91, 9229. 31251561 10.1021/acs.analchem.9b02091

