# Supplementary material for: Single‐Entity Electrochemistry of N‐Doped Graphene Oxide Nanostructures for Improved Kinetics of Vanadyl Oxidation
Source: Small. 2024 Nov 16;21(3):2405220. doi: 10.1002/smll.202405220 (PMC11753488; doi:10.1002/smll.202405220)
Supplement: Supplementary file 1 — Supporting Information [file SMLL-21-2405220-s001.pdf]

NANO · MICRO
small

Supporting Information

for *Small*, DOI 10.1002/smll.202405220

Single-Entity Electrochemistry of N-Doped Graphene Oxide Nanostructures for Improved Kinetics of Vanadyl Oxidation

Maida Aysla Costa de Oliveira, Marc Brunet Cabré, Christian Schröder, Hugo Nolan, Filippo Pota, James A. Behan, Frédéric Barrière, Kim McKelvey and Paula E. Colavita**

Supplementary Information

Single-entity electrochemistry studies of N-doped graphene oxide nanostructures for improved kinetics of vanadyl oxidation

Maida Aysla Costa de Oliveira,[1†] Marc Brunet-Cabré,[1†] Christian Schröder,[1] Hugo Nolan,[1] Filippo Pota,[1] James A. Behan,[2] Frédéric Barrière,[2] Kim McKelvey[3] and Paula E. Colavita[11]*

[1] School of Chemistry, Trinity College Dublin, Dublin 2, Ireland.

[2] Univ Rennes, CNRS, Institut des Sciences Chimiques de Rennes – UMR 6226, F-35000 Rennes, France.

[3] MacDiarmid Institute for Advanced Materials and Nanotechnology, School of Chemical and Physical Sciences, Victoria University of Wellington, Wellington 6012, New Zealand

[1] Corresponding authors: colavitp@tcd.ie and kim.mckelvey@vuw.ac.nz
† Authors contributed equally to this work.

Table S1. Comparison of anodic peak potentials ($E_{p,a}$), anodic current onset potentials (E_{onset}), current densities and energy efficiencies reported in literature studies of vanadyl oxidation processes.

Graphene type	E_{onset} (V vs. Ag/AgCl)	$E_{p,a}$ (V vs. Ag/AgCl)	Scan rate (V s^{-1})	VO^{2+}	H_2SO_4	Ref.
N/O-doped Graphene	0.80	0.95	0.05	0.01 M	1.5 M	This work
Graphene/multi-walled	0.90	1.00	0.005	0.05 M	3.0 M	[1]
Graphene Oxide	0.70	0.90	0.02	0.10 M	2.0 M	[2]
Graphene Oxide	0.80	1.00	0.01	0.10 M	3.0 M	[3]
Graphene Oxide	0.70	1.20	0.02	0.05 M	1.0 M	[4]
N-doped graphene	0.70	1.20	0.02	0.05 M	1.0 M	[5]
N-doped graphene	0.75	0.95	0.05	1.00 M	3.0 M	[6]
N-doped graphene	0.80	1.10	0.005	0.10 M	3.0 M	[7]
N-doped rGO	0.85	1.10	0.05	0.10 M	4.0 M	[8]
HOPG	0.90	1.15	0.02	0.10 M	2.0 M	[8]
GC	1.00	1.15	0.05	0.01 M	1.5 M	This work

Graphene type	Current density (mA cm^{-2})	Energy efficiency (%)	VO^{2+}	H_2SO_4	Ref.
Nanostructured graphene	300	93.9	1.0 M	3.0 M	[7]
N-doped carbon felt	300	73.6	1.0 M	3.0 M	[9]
N-doped mesoporous graphene	200	75.0	0.015 M	3.0 M	[10]
GO carbon felt	100	74.9	0.05 M	1.0 M	[4]
O-modified carbon paper	140	78.1	0.05 M	3.0 M	[11]
O-modified carbon cloth	200	90.8	1.0 M	3.0 M	[12]
Carbon paper	100	85.1	1.7 M	4.2 M	[13]
Carbon felt	40	82.3	1.5 M	4.0 M	[2]
Graphite felt	50	76.2	0.1 M	3.0 M	[14]

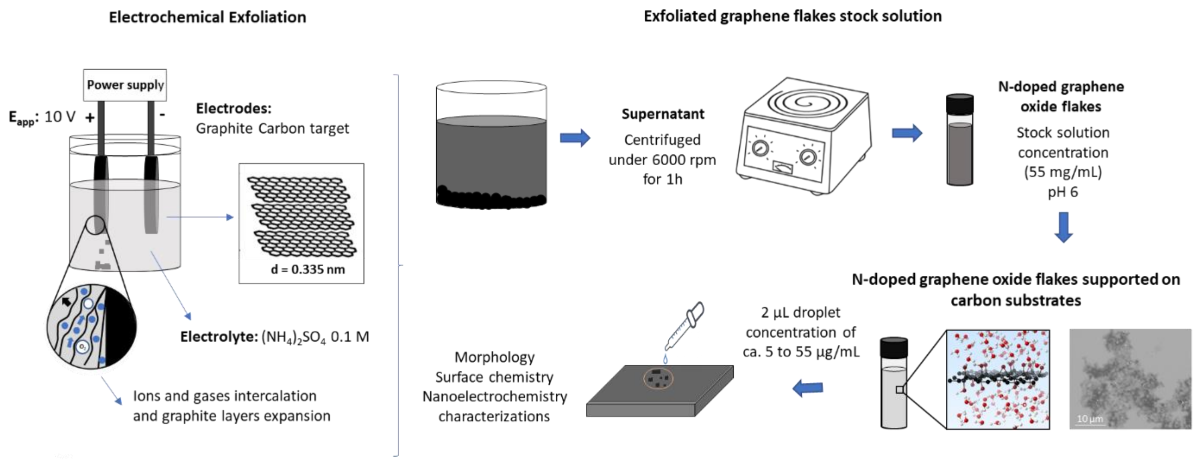

Figure S1. Scheme showing electrochemical exfoliation conditions used to produce graphene nanostructures with oxygen and nitrogen functionalities and electrode preparation method for characterization of morphology, surface chemistry and electrochemical performance.

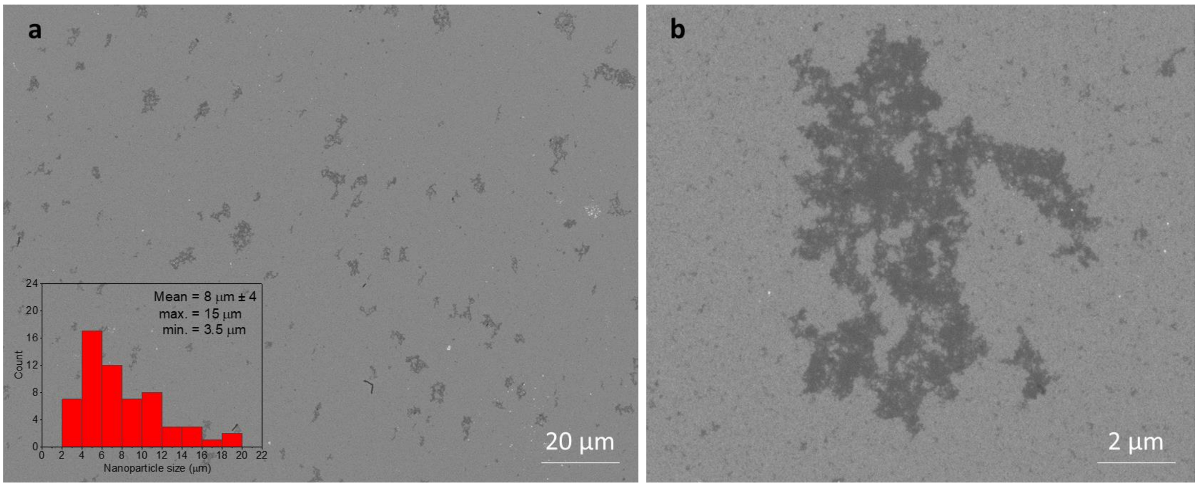

Figure S2. SEM images of drop-cast N-doped GO flakes. The inset shows the size distribution calculated based on analysis of SEM images.

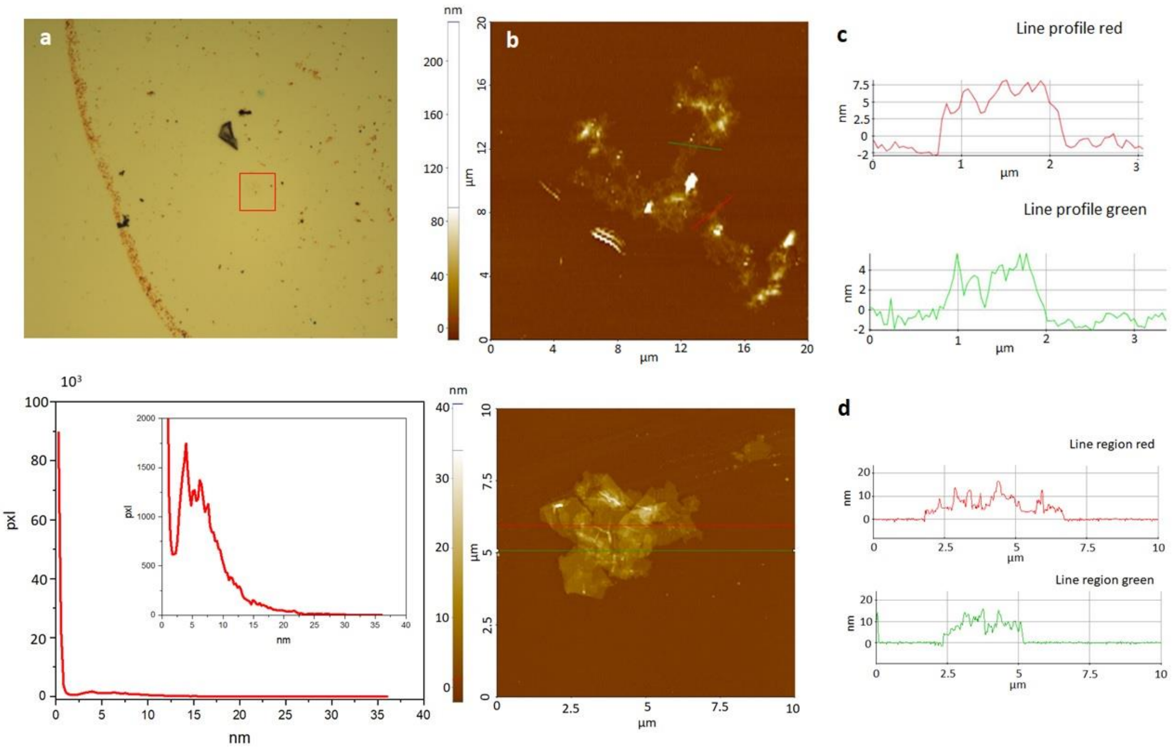

Figure S3. (a) Optical microscopy image of drop-cast GO nanostructures on a carbon substrate electrode, showing the "coffee ring" feature that defines the GO-modified regions. The red square indicates the area chosen for further characterization. (b) AFM height image of a GO flake and (c) height profiles obtained at the GO-substrate edges. (d) Additional details of height distribution for the drop-cast GO nanostructure in Figure 2b. The majority of the flake surface displays height comparable to that of the step edges discussed in the text; this can be seen from a height distribution over the entire image (left) or from examples of full line profiles across the GO flake (right).

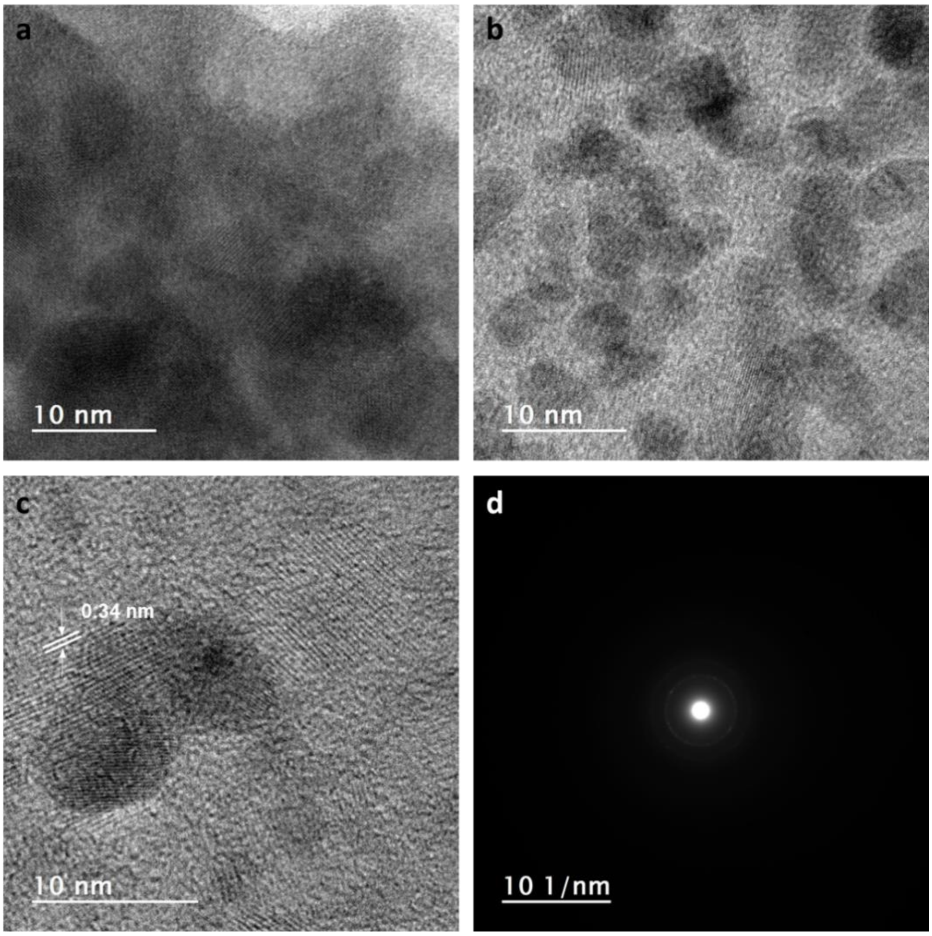

Figure S4. TEM images of N-doped GO flakes over three different regions (a), (b), and (c). (d) Selected area diffraction (SEAD) pattern of N-doped GO flakes.

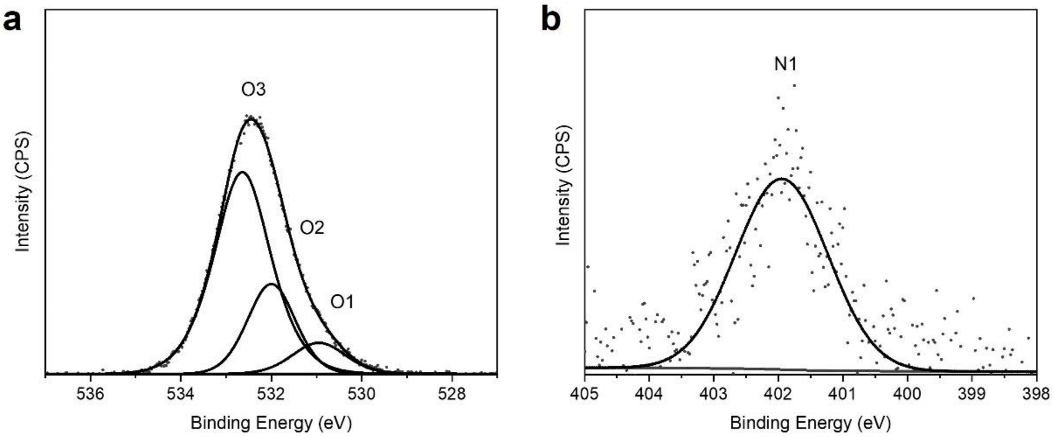

Figure S5. X-ray photoelectron spectroscopy (XPS) characterization of nanostructured graphene flakes. The figure shows high-resolution spectra in the (a) O 1s and (b) N 1s regions and their best-fits.

Table S2. Results from analysis of best-fits of high-resolution XPS spectra of GO on Si wafers.

**Components**	**Peak position**	**Atomic %**	**O/C ratio**	**N/C ratio**
C 1s	284.5	51.5%		
O 1s	232.5	42.7%	65%	
N 1s	401.9	1.3%		2.5%
Si 2p of SiO_2[15]	103.6	4.5%		

**C 1s**	**BE (eV)**	**%**	**O 1s**	**BE (eV)**	**%**	**N1s**	**BE (eV)**	**%**
C1	284.5	44.29	O1	531.0	9.2	N1	401.9	100
C2	285.1	13.61	O2	532.0	24.0			
C3	286.7	29.48	O3	532.6	66.8			
C4	288.4	11.16						
π-π*	290.2	1.46						

Analysis and deconvolution of the C 1s peak obtained with 5 components (see main text) corresponding to C1 as C-C (C-sp^2), C2 as C-sp^3 (C-NH_2), C3 as C-O and C=N, C4 (O-C=O), and π-π* shake-up. The O 1s region was fit with three components corresponding to O=C (O1), C-O (O2), and C-OH (O3). The N 1s spectrum was best-fit with only one peak for all area calculations.

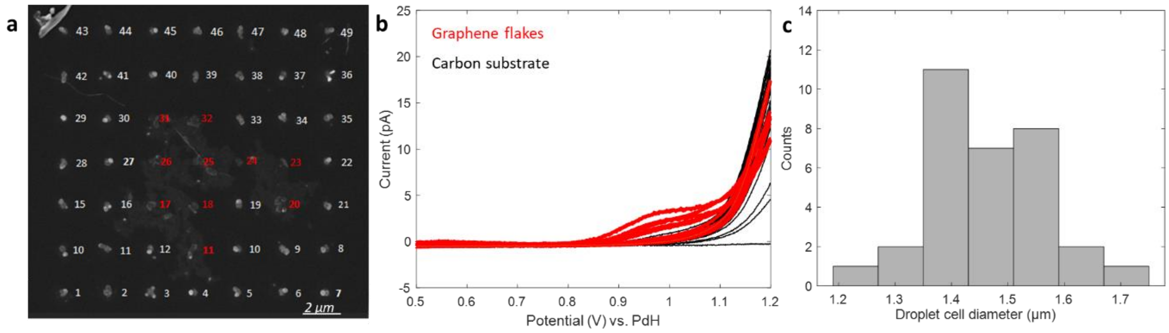

Figure S6. (a) SEM image of the N-doped GO flake at the thin-film carbon substrate electrode after SECCM measurements; each probed location is indexed based on the order of measurement in the raster grid pattern (#1-#49); colour-coding indicates a probed GO (red) or substrate (white) location based on the SEM contrast. (b) LSV obtained at the 49 points in the grid shown in (a); points corresponding to N-doped GO probed locations are shown in red, while the substrate is shown in black. Note that points #1-#3 show anomalously low currents due to droplet instabilities during the first nanopipette approaches. (c) Histogram of droplet cell diameters measured from SEM analysis of electrolyte residues.

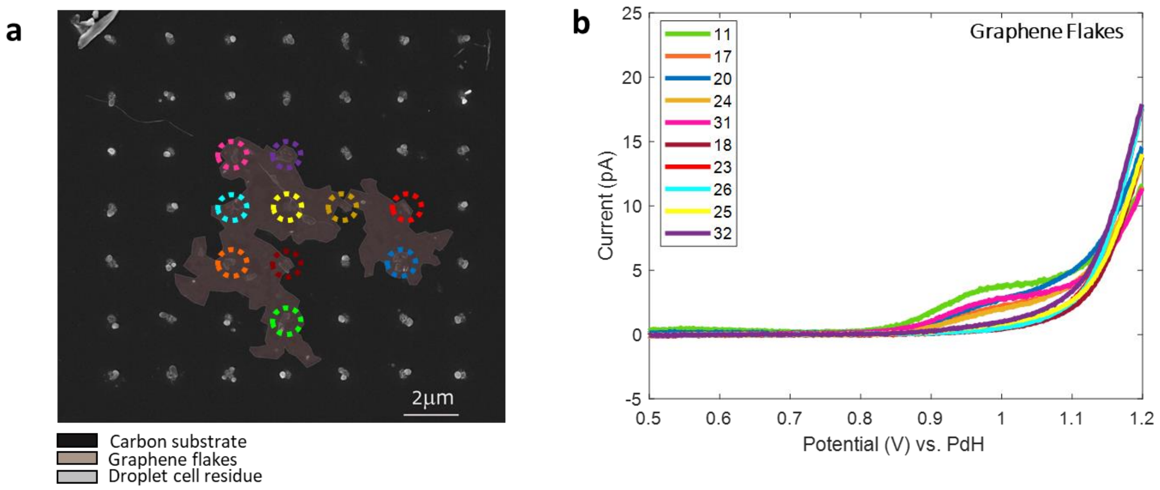

Figure S7. (a) SEM image of the N-doped GO flake at the thin-film carbon substrate electrode after SECCM measurements shown in Figure S6a above, with each probed GO location circled in a different color to facilitate reference to the corresponding LSV curves. (b) LSV obtained at the 10 points in the grid shown in (a) corresponding to N-doped GO probed locations; colours correspond to those of circled locations in panel (a), while curve indexes can be mapped to those in Figure S6a.

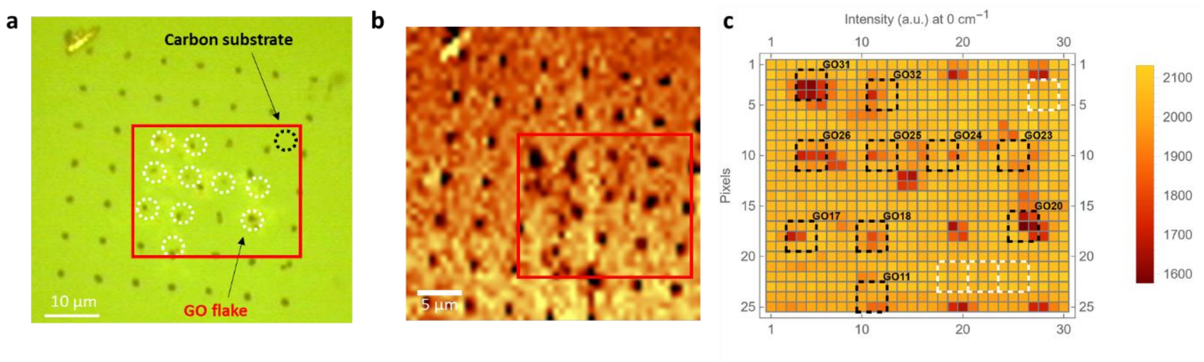

Figure S8. (a) Optical image and (b) Raman chemical image (25×30 pixels) at the Rayleigh intensity showing the points probed in the SECCM grid. (c) Intensity at 0 cm^{-1} showing image pixels selected for Raman analysis. Dashed rectangles indicate regions used for the calculation of the average Raman spectra of the carbon thin film substrate (white) and of GO and substrate together (black); labels indicate the corresponding point index in the SECCM grid as shown in **Figure S6**.

Table S3. Results from analysis of Raman spectra at locations shown in Figure S8. The anodic currents recorded at the same points are shown tabulated for comparison.

GO grid point	Area (arb. U.)	D/G (by height)	Current @0.95 V (pA)
#11	2106	1.4	3.2
#17	2973	1.1	1.6
#18	1732	1.0	0.30
#20	3209	1.3	2.0
#23	3778	1.2	0.3
#24	1274	1.7	1.4
#25	617	-	0.8
#26	3324	1.5	0.3
#31	5715	1.5	2.2
#32	1432	2.1	0.6
Average	2944	1.5	-

Text S1. Details of LSV simulations

A 2D axisymmetric model of the pipette probe in contact with the working electrode was implemented using COMSOL Multiphysics v.5.4. The pipette was approximated as a truncated hollow cone (**Figure 5a** in the main text) with parameters in **Table S4** [16]. The cone angle was set at 12° [16-18], the aperture radius r_{ap} was assumed to be equal to that of the electrolyte residues and the meniscus drop equal to r_{ap}. Boundary coordinates and boundary conditions are reported in **Table S5.** Initial concentrations and scan rate were set to match the conditions of SECCM experiments. The time-dependent simulation was solved with an intermediate time step of 0.1 s over a triangular mesh. The mass-transport of species in solution was simulated using Fick's laws. For the E-step, the flux of electrochemically active species (VO^{2+} and VO_2^+) at the electrode surface/s was defined by Butler-Volmer kinetics according to [19]:

$$k_{f,j} = k_j^0 e^{\left(\frac{(1-\alpha)F\eta}{RT}\right)} \qquad \text{(eq. 1)}$$

$$k_{b,j} = k_j^0 e^{\left(\frac{-\alpha F\eta}{RT}\right)} \qquad \text{(eq. 2)}$$

where F is Faraday's constant, α is the symmetry factor, R is the ideal gas constant, T is temperature, k_j^0 is the standard heterogeneous electron transfer rate constant in cm s^{-1} at either substrate (j = *sub*) or defect (j = *def*) surfaces, and $\eta = E - E^{0'}$ is the overpotential. Simulations were generated with parametric sweeps as discussed in the main text with conditions summarized in **Table S6.** Best-fit parameters were identified by locating the minimum of the sum of square errors (SSE). Uncertainties on best-fit k_{def}^0 values were obtained by identifying the uniqueness range, defined by a 30% increase in SSE relative to the best-fit minimum [20, 21]. Note that uncertainty determinations arise from 2D projections of a 3D error space, thus in each case the range reported is the interval that encompasses the lowest and highest value over the (k^0, E^0) and (k^0, r) projections, to accurately reflect the spread of values around the best-fit minima of the SSE.

Table S4: Summary of parameters used in finite element simulations.

Name	Value	Units	Description
r_{ap}	0.7	µm	Radius of the pipette aperture.
h_{pip}	100	µm	Height of the pipette probe in the model.
θ	12	°	Cone angle of the pipette probe.
v	0.050	$V\ s^{-1}$	Scan rate
$\boldsymbol{\alpha}$	0.5	-	Butler-Volmer Transfer coefficient
$\boldsymbol{D_{VO^{2+}}}$	$2.1 \cdot 10^{-6}$	$cm^2\ s^{-1}$	Diffusion coefficient of VO^{2+}
$\boldsymbol{D_{VO_2{}^+}}$	$2.8 \cdot 10^{-6}$	$cm^2\ s^{-1}$	Diffusion coefficient of $VO_2{}^+$
$\boldsymbol{c0_{VO^{2+}}}$	15.0	mM	Initial concentration of VO^{2+} in the electrolyte solution domain
$\boldsymbol{c0_{VO_2{}^+}}$	0	mM	Initial concentration of $VO_2{}^+$ in the electrolyte solution domain
$\boldsymbol{T}$	298	K	Temperature
$\boldsymbol{E^{0\prime}_{sub}}$	0.869	V	Formal potential (vs PdH) of the VO^{2+}/ $VO_2{}^+$ redox couple at the thin-film carbon substrate (vs PdH)

Table S5: Cylindrical coordinates (r and z) used to describe each boundary and domain in the finite element simulation and corresponding flux and concentration conditions. c_i refers to the concentration of i species; $k_{f,j}$ and $k_{b,j}$ refer to the forward and backward Butler Volmer rate constants, respectively, at the active defect (j = def) or at the carbon thin-film substrate (j = sub).

Description	Coordinates $\boldsymbol{z}$	Coordinates $\boldsymbol{r}$	Boundary condition
Axis of Symmetry	$0 \leq \boldsymbol{z} \leq h_{pip}$	0	–
Bulk solution	h_{pip}	$0 \leq \boldsymbol{r} \leq r_{ap} + h_{pip} \tan\theta$	$c_{VO^{2+}} = c0_{VO^{2+}}$<br>$c_{VO_2{}^+} = c0_{VO_2^+}$
Pipette wall	$r_{ap} \leq \boldsymbol{z} \leq h_{pip}$	$r_{ap} \leq \boldsymbol{r} \leq r_{ap} + \boldsymbol{z} \tan\theta$	J = 0<br>(No flux)
Meniscus/air interface	$0 \leq \boldsymbol{z} \leq r_{ap}$	r_{ap}	J = 0<br>(No flux)
Electrode Surface	0	$0 \leq \boldsymbol{r} \leq r_{def}$	$\nabla c_{VO^{2+}} = -k_{f,def} c_{VO^{2+}} + k_{b,def} c_{VO_2^+}$<br>$\nabla c_{VO_2^+} = -\nabla c_{VO^{2+}}$
	0	$r_{def} \leq \boldsymbol{r} \leq r_{ap}$	$\nabla c_{VO^{2+}} = -k_{f,sub} c_{VO^{2+}} + k_{b,sub} c_{VO_2^+}$<br>$\nabla c_{VO_2^+} = -\nabla c_{VO^{2+}}$

Table S6. Conditions chosen for parametric sweeps of the electrochemical response at surfaces.

Name	Value	Units	Description
	**At carbon thin-film substrates**		
$\boldsymbol{k^0_{sub}}$	$k^0 = a_1 \cdot r^{(n-1)}$<br>$a_1 = 1 \cdot 10^{-7}$ $r = 1.166$ $n = 1{:}76$	cm s^{-1}	Heterogeneous rate constant at the thin-film carbon substrate
$\boldsymbol{E^{0\prime}_{sub}}$	0.65 : 1.10<br>ΔV = 0.003	V	Formal potential of the VO^{2+}/ VO_2^+ redox couple at the thin-film carbon substrate
	**At N-doped GO active defect sites**		
$\boldsymbol{r_{def}}$	r_{ap}/b, b = 100 to 1260<br>$b = a_1 \cdot r^{(n-1)}$<br>$a_1 = 100$ $r = 1.259$ $n = 1{:}12$	µm	Radius of the active defect area at GO flakes, expressed as a fraction of the pipette aperture radius.
$\boldsymbol{k^0_{def}}$	$k^0 = a_1 \cdot r^{(n-1)}$<br>$a_1 = 1 \cdot 10^{-2}$ $r = 1.585$ $n = 1{:}26$	cm s^{-1}	Heterogeneous rate constant at active defect regions on GO flakes
$\boldsymbol{E^{0\prime}_{def}}$	0.86 : 0.97<br>ΔV = 0.010	V	Formal potential of the VO^{2+}/ VO_2^+ redox couple at active defects

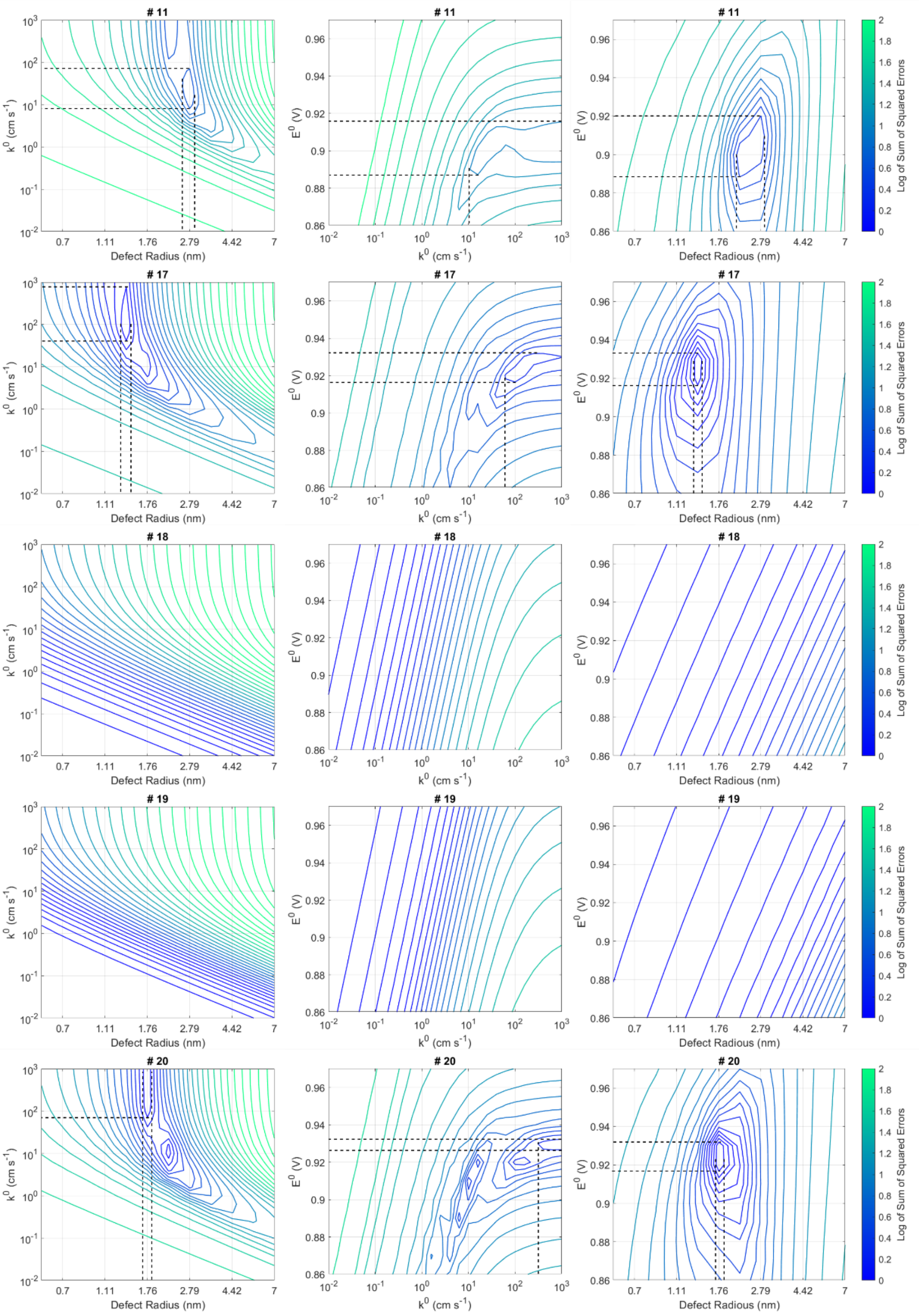

Figure S9. Contour plots near the SSE minimum for all ten LSV obtained at GO sub-domains; contour lines indicate +30% increases in SSE relative to the preceding contour line. The grid point fitted in each case is indicated at the top of the panel. Continues in the next pages.

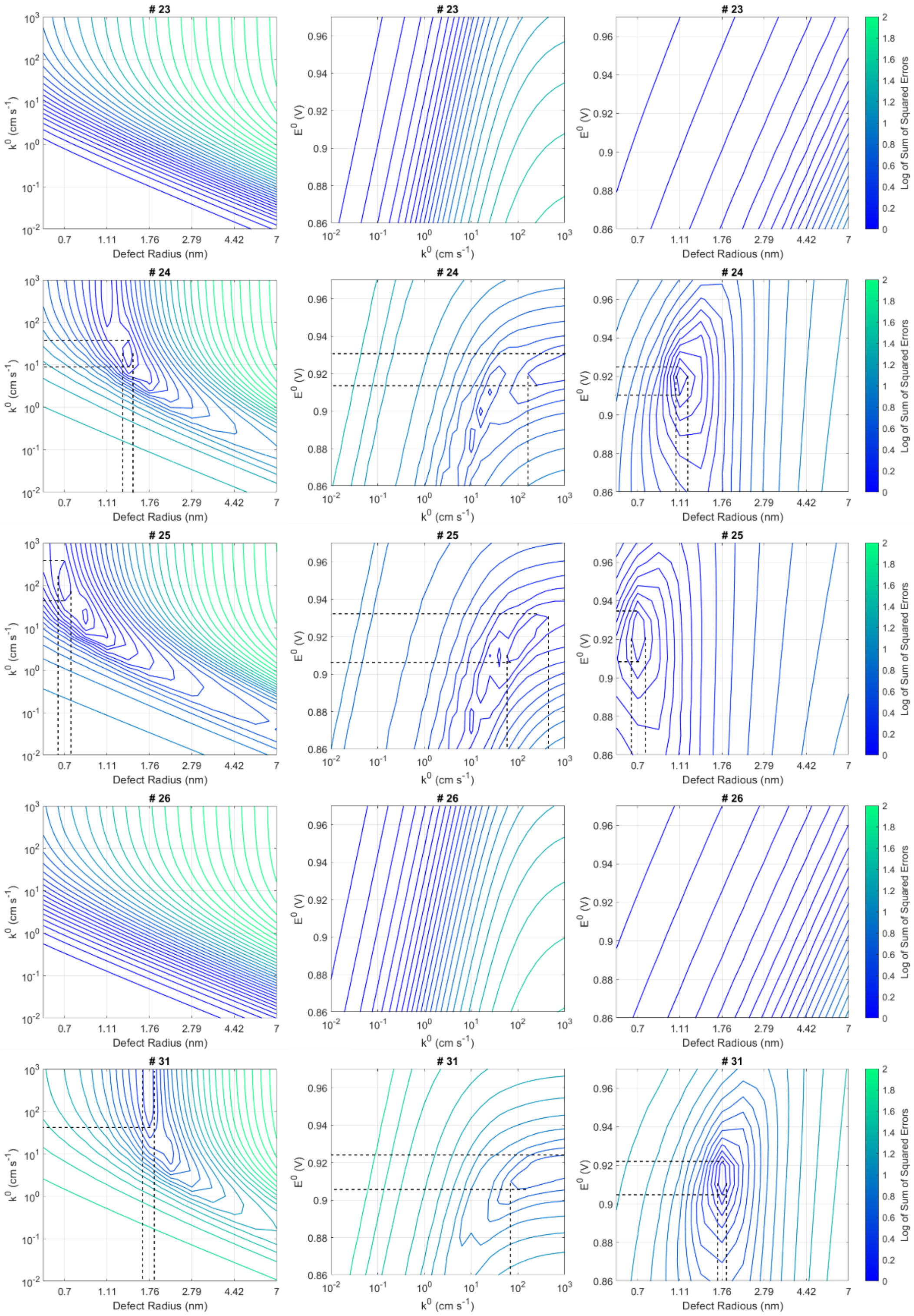

23
24
25
26
31
k0 (cm s-1)
E0 (V)
Defect Radius (nm)
Defect Radious (nm)
Log of Sum of Squared Errors

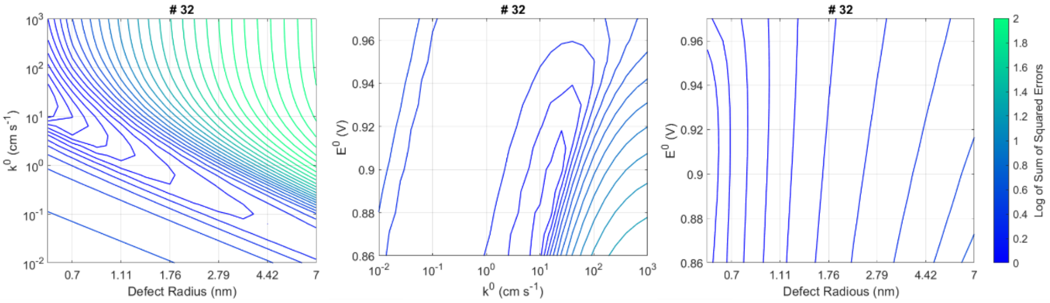

32
k^0 (cm s^{-1})
Defect Radius (nm)
0.7
1.11
1.76
2.79
4.42
7
32
E^0 (V)
k^0 (cm s^{-1})
32
E^0 (V)
Defect Radious (nm)
Log of Sum of Squared Errors

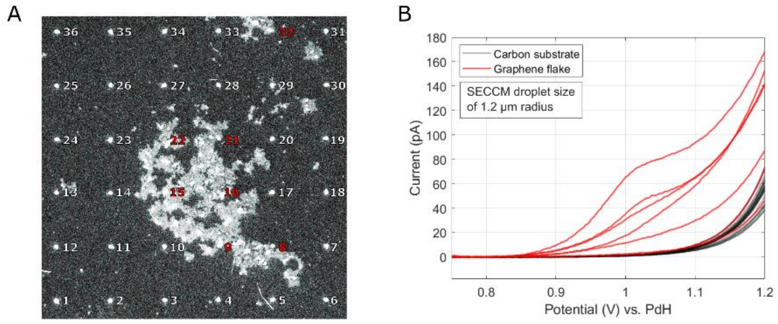

Figure S10. (a) SEM image of an N-doped GO flake at a thin-film carbon substrate electrode after SECCM measurements; each probed location is indexed based on the order of measurement in the raster grid pattern; colour-coding indicates a probed GO (red) or substrate (white) location based on the SEM contrast. The brightness and contrast in the SEM image have been modified to facilitate identification of the GO flake outline. (b) LSV obtained at the points in the grid shown in (a); points corresponding to N-doped GO probed locations are shown in red, while the substrate is shown in black. LSV were obtained at 5.0 V s^{-1} using 15.0 mM VO^{2+} in 0.150 M H_2SO_4.

Text S2. Additional evidence supportive of model assumptions

The model adopted for the simulation of the current response assumes a disk shape geometry, with no intra/interparticle porosity associated with either the internal structure of the GO flake probed or the presence of agglomerates. Neglecting the role played by the microscopic area of the electrode has been shown in the literature to potentially lead to misinterpretations of activity as depletion of redox species in voids can lead to current contributions at low overpotential in voltammetry studies at macroelectrodes [22]. However, it is possible to exclude contributions from porosity based on an analysis of current magnitudes and z-positioning of the SECCM probe, as below.

It is possible to estimate the maximum faradaic charge (Q_{void}) that could be physically obtained as a result of depletion within a pore volume (V_{void}) that is equivalent to the entire volume of the probed flake. This would represent an upper boundary value for the faradaic charge in the case of porosity, that can then be compared to the experimental charges associated with the current plateaus in Figure 3.

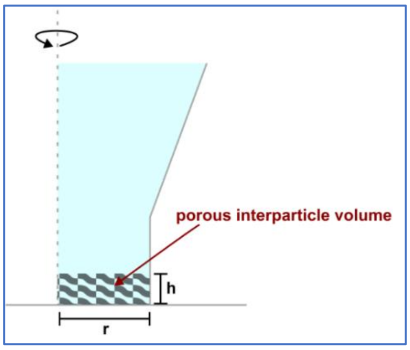

Assuming a cylindrical void volume generated by GO stacked layers with height h = 5 nm, a radius equal to the pipette aperture r = 0.7 µm, and a concentration of redox-active species of 15 mM, then:

$$V_{void} = \pi r^2 h = 7.7 \times 10^{-21}\ \mathrm{m}^3$$

$$Q_{void} = V_{void}\,[VO^{2+}] \cdot n \cdot F = 1.1 \times 10^{-14}\ \mathrm{C}$$

The faradaic charges passed in the current plateaus in Figure 3 are much larger than this. Active GO-contacted locations yield currents of 1-3 pA over 0.8-1.1 V at 50 mV/s, equivalent to integrated charges ranging from 0.5 to 1.2×10^{-11} C. Such values are around three orders of magnitude larger than those that could conceivably result from vanadyl depletion in GO flake voids at contacted locations. This remains valid even if one allows for the flake to swell at the contact point, given that interlayer spacing increases are reported to be 1 nm or less in water [23]. A current plateau i_{est} associated with the void depletion of redox species would be ca. ×1000 smaller than the ones observed experimentally:

$$i_{est} \cong \frac{Q_{void}\ 0.050\ V/s}{1.1 - 0.8\ V} \cong 0.002\ pA$$

For i_{est} to become comparable to the experimental plateaus, the void volume would have to be extremely large and result from GO agglomerates/deposits that are much larger than any topographic irregularities observed in the SEM/AFM characterization of GO flakes. The presence of such agglomerates would also become readily apparent from Raman intensities at probed locations which, on the contrary, do not show any evidence of thickness variations across GO-contacted locations. Therefore, the experimental current plateaus cannot arise from to depletion in a porous intraparticle or agglomerate volume, as these would need to be at least ~100-1000 times larger/thicker than the observed GO flakes to yield the experimentally observed currents at low overpotentials. Mass transport of redox species outside the flake volume must contribute to achieving the currents observed so that the depletion layer extends much farther from the electrode than the height of the GO flakes, at which point the effect of porosity is unlikely to influence the current response. Such conditions justify the assumption of a planar electrode surface in the simulations in Figure 5.

Finally, the presence of such large agglomerates is inconsistent with experimental values of the piezo-positioner that controls the height of the SECCM nanopipette probe. **Figure S11** shows z-height position of the engaged nanopipette for the 7×7 grid in the form of a heat map and a cluster plot. It is evident that z-height values at GO-contacted and substrate-contacted locations cannot be distinguished. This supports the conclusion that the experimental currents cannot arise from large agglomerates.

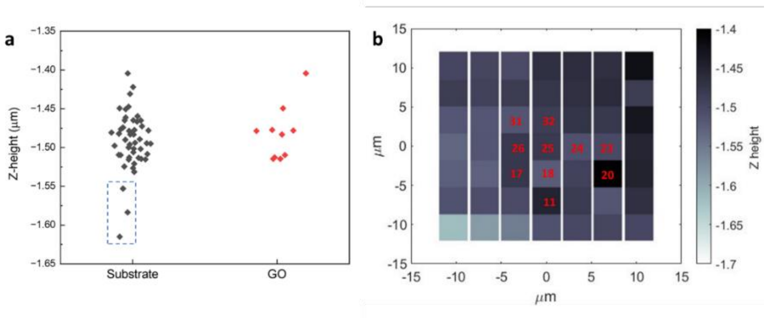

Figure S11. Z-height position of the engaged nanopipette for the 7×7 grid simulated in Figure 5 in the form of (a) cluster plot and (b) heat map; the GO-contacted locations are indicated in red with the pt# corresponding to the notation used in the discussion. The dashed frame indicates the initial three points in the grid, which are typically affected by droplet instabilities during the first approaches in a grid.

References

[1] M. AlNahyan, I. Mustafa, A. Alghaferi, F. Almarzooqi, Porous 3D graphene/multi-walled carbon nanotubes electrodes with improved mass transport and kinetics towards VO^{2+}/VO_2^{+} redox couple, Electrochim. Acta, 385 (2021) 138449.

[2] A. Sankar, I. Michos, I. Dutta, J. Dong, A.P. Angelopoulos, Enhanced vanadium redox flow battery performance using graphene nanoplatelets to decorate carbon electrodes, J. Power Sources, 387 (2018) 91-100.

[3] M. Park, I.Y. Jeon, J. Ryu, J.B. Baek, J. Cho, Exploration of the Effective Location of Surface Oxygen Defects in Graphene-Based Electrocatalysts for All-Vanadium Redox-Flow Batteries, Adv. Energy Mater., 5 (2015) 1401550.

[4] D.O. Opar, R. Nankya, J. Lee, H. Jung, Three-dimensional mesoporous graphene-modified carbon felt for high-performance vanadium redox flow batteries, Electrochim. Acta, 330 (2020) 135276.

[5] D.O. Opar, R. Nankya, J. Lee, H. Jung, Assessment of three-dimensional nitrogen-doped mesoporous graphene functionalized carbon felt electrodes for high-performance all vanadium redox flow batteries, Appl. Surf. Sci., 531 (2020) 147391.

[6] J. Jin, X. Fu, Q. Liu, Y. Liu, Z. Wei, K. Niu, J. Zhang, Identifying the Active Site in Nitrogen-Doped Graphene for the VO^{2+}/VO_2^{+} Redox Reaction, ACS Nano, 7 (2013) 4764-4773.

[7] S. Bellani, L. Najafi, M. Prato, R. Oropesa-Nuñez, B. Martín-García, L. Gagliani, E. Mantero, L. Marasco, G. Bianca, M.I. Zappia, C. Demirci, S. Olivotto, G. Mariucci, V. Pellegrini, M. Schiavetti, F. Bonaccorso, Graphene-Based Electrodes in a Vanadium Redox Flow Battery Produced by Rapid Low-Pressure Combined Gas Plasma Treatments, Chem. Mater., 33 (2021) 4106-4121.

[8] X. Michel Myures, S. Suresh, Nanofluidic electrolyte based on nitrogen doped reduced graphene oxide as an electrocatalyst for VO_2^{+}/VO^{2+} in vanadium redox flow battery, J. Energy Storage, 58 (2023) 106387.

[9] K. Zhang, C. Yan, A. Tang, Interfacial co-polymerization derived nitrogen-doped carbon enables high-performance carbon felt for vanadium flow batteries, J. Mater. Chem. A, 9 (2021) 17300-17310.

[10] J. Ji, C. Noh, M. Shin, S. Oh, Y. Chung, Y. Kwon, D.-H. Kim, Vanadium redox flow batteries using new mesoporous nitrogen-doped carbon coated graphite felt electrode, Appl. Surf. Sci., 611 (2023) 155665.

[11] T. Liu, X. Li, C. Xu, H. Zhang, Activated Carbon Fiber Paper Based Electrodes with High Electrocatalytic Activity for Vanadium Flow Batteries with Improved Power Density, ACS Appl. Mater. Interfaces, 9 (2017) 4626-4633.

[12] X.L. Zhou, T.S. Zhao, Y.K. Zeng, L. An, L. Wei, A highly permeable and enhanced surface area carbon-cloth electrode for vanadium redox flow batteries, J. Power Sources, 329 (2016) 247-254.

[13] J.-M. Jeong, K.I. Jeong, J.H. Oh, Y.S. Chung, S.S. Kim, Stacked carbon paper electrodes with pseudo-channel effect to improve flow characteristics of electrolyte in vanadium redox flow batteries, Appl. Mater. Today, 24 (2021) 101139.

[14] Y. Lv, Y. Yang, J. Gao, J. Li, W. Zhu, L. Dai, Y. Liu, L. Wang, Z. He, Controlled synthesis of carbon nanonetwork wrapped graphite felt electrodes for high-performance vanadium redox flow battery, Electrochim. Acta, 431 (2022) 141135.

[15] D.S. Jensen, S.S. Kanyal, N. Madaan, M.A. Vail, A.E. Dadson, M.H. Engelhard, M.R. Linford, Silicon (100)/SiO_2 by XPS, Surf. Sci. Spectra, 20 (2013) 36-42.

[16] D. Perry, D. Momotenko, R.A. Lazenby, M. Kang, P.R. Unwin, Characterization of Nanopipettes, Anal. Chem., 88 (2016) 5523-5530.

[17] G. Jayamaha, M. Maleki, C.L. Bentley, M. Kang, Practical guidelines for the use of scanning electrochemical cell microscopy (SECCM), Analyst, 149 (2024) 2542-2555.

[18] S. Zhang, M. Li, B. Su, Y. Shao, Fabrication and Use of Nanopipettes in Chemical Analysis, Annu. Rev. Anal. Chem., 11 (2018) 265-286.

[19] D. Li, C. Batchelor-McAuley, R.G. Compton, Some thoughts about reporting the electrocatalytic performance of nanomaterials, Appl. Mater. Today, 18 (2020) 100404.

[20] J.N. Hilfiker, N. Singh, T. Tiwald, D. Convey, S.M. Smith, J.H. Baker, H.G. Tompkins, Survey of methods to characterize thin absorbing films with Spectroscopic Ellipsometry, Thin Solid Films, 516 (2008) 7979-7989.

[21] B. Singh, A. Diwan, V. Jain, A. Herrera-Gomez, J. Terry, M.R. Linford, Uniqueness plots: A simple graphical tool for identifying poor peak fits in X-ray photoelectron spectroscopy, Appl. Surf. Sci., 387 (2016) 155-162.

[22] C. Punckt, M.A. Pope, I.A. Aksay, High Selectivity of Porous Graphene Electrodes Solely Due to Transport and Pore Depletion Effects, J. Phys. Chem. C, 118 (2014) 22635-22642.

[23] A. Iakunkov, A.V. Talyzin, Swelling properties of graphite oxides and graphene oxide multilayered materials, Nanoscale, 12 (2020) 21060-21093.
